# A Proton‐Intercalation Pathway Realizes Long‐Life Manganese‐Ion Hybrid Batteries With Layered KV_3_O_8_

**DOI:** 10.1002/advs.74524

**Published:** 2026-02-23

**Authors:** Sangki Lee, Hyungjin Lee, Jangwook Pyun, Hyeonjun Lee, Ki‐Min Roh, Kang Taek Lee, Seung‐Tae Hong, Xiaoyan Jin, Incheol Jeong, Seong‐Ju Hwang, Munseok S. Chae

**Affiliations:** ^1^ Department of Nanotechnology Engineering Pukyong National University Busan Republic of Korea; ^2^ Department of Materials Science and Engineering Yonsei University Seoul Republic of Korea; ^3^ Department of Energy Science and Engineering DGIST Daegu Republic of Korea; ^4^ Resources Utilization Research Center Korea Institute of Geoscience and Mineral Resources Daejeon Republic of Korea; ^5^ Department of Mechanical Engineering Korea Advanced Institute of Science and Technology Daejeon Republic of Korea; ^6^ KAIST Graduate School of Green Growth & Sustainability Daejeon Republic of Korea; ^7^ Department of Applied Chemistry University of Seoul Seoul Republic of Korea; ^8^ Department of Battery Engineering Yonsei University Seoul Republic of Korea

**Keywords:** aqueous electrolytes, cathode materials, hybrid batteries, KV_3_O_8_

## Abstract

Rechargeable aqueous manganese‐based batteries offer a low‐cost, high‐safety, and promising solution to meet the growing demand for large‐scale energy storage systems. However, since aqueous manganese‐ion batteries (AMIBs) are still in the early stages of development, discovering high‐performance cathode materials is critical for enabling future commercialization. In this study, we introduce monoclinic KV_3_O_8_ as a highly stable and unprecedented cathode material for AMIBs. KV_3_O_8_ has a layered structure with an interlayer spacing of approximately 7.63 Å (d_001_), which facilitates the reversible intercalation and deintercalation of cations. This structural feature ensures excellent long‐term cycling stability (88.0% capacity retention after 3600 cycles) and outstanding rate capability. By integrating diffusion path and barrier calculations with X‐ray photoelectron spectroscopy, ex situ X‐ray absorption spectroscopy, ex situ X‐ray diffraction, Fourier‐transform infrared spectroscopy, and Raman spectroscopy, we identify both Mn^2+^ ions and protons as active charge carriers. Furthermore, the formation of a Mn(OH)_2_ layer on the cathode surface during discharge suggests that protons predominantly govern the charge storage mechanism. This study provides critical insights into the design of advanced manganese ion cathode materials and represents a significant step toward the practical realization of AMIBs.

## Graphical abstract

This is the first study to demonstrate layered monoclinic KV_3_O_8_ as a highly stable cathode material for aqueous Mn‐based hybrid batteries, exhibiting excellent cycling stability with 88.0% capacity retention after 3600 cycles. The proton storage mechanism is systematically elucidated through comprehensive spectroscopic analyses, further supported by DFT calculations.

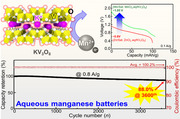

## 1 Introduction

The increasing demand for electric vehicles (EVs) and large‐scale energy storage technologies has necessitated advancements in energy storage systems. Among the currently developed technologies, lithium‐ion batteries (LIBs) have been widely adopted in EVs, portable electronics, and energy storage systems owing to their relatively mature technology and high energy density [1]. However, LIBs face limitations because of the scarce lithium resources and the safety concerns and high costs associated with these batteries, underscoring the urgent need for next‐generation energy storage technologies. To meet the growing demand, the development of batteries with high safety, low cost, high energy density, and enhanced power performance is essential. Compared to conventional non‐aqueous LIBs, aqueous rechargeable metal‐ion batteries have garnered significant research interest owing to their enhanced safety characteristics, as they mitigate the risk of fire hazards posed by organic electrolytes [2, 3, 4].

Owing to these advantages, extensive research has been dedicated to the development of aqueous metal‐ion batteries [5, 6], including aqueous aluminum‐ion batteries [7], aqueous iron‐ion batteries [8, 9, 10], and aqueous zinc‐ion batteries [11, 12, 13]. Because aluminum is trivalent, aluminum‐ion batteries are regarded as a promising next‐generation charge storage technology [7]. However, this system does not effectively use an aluminum metal anode, although aluminum exhibits a lower redox potential (−1.66 V vs. SHE) than zinc. When aluminum reacts with water, an insulating aluminum oxide layer forms, preventing its reversible operation as an anode. As a result, aluminum‐ion batteries function as a rocking‐chair‐type system, which inherently limits their voltage output. Recently, iron‐ion batteries have also been explored as a low‐cost aqueous energy storage system [8, 9, 10]. The iron metal anode can reversibly operate in an aqueous electrolyte. However, its high redox potential (−0.44 V vs. SHE) is an intrinsic limitation to this system. Among aqueous metal‐ion batteries, zinc‐based systems have received the greatest research attention [11, 12, 13]. The zinc metal anode undergoes reversible deposition–dissolution processes with a low redox potential of −0.76 V versus SHE, making it highly suitable for aqueous battery applications. Leveraging this advantage, numerous cathode materials have been reported so far. However, many of these materials still exhibit suboptimal operating voltages. For instance, vanadium‐based cathodes typically operate at ∼1 V, while manganese oxide cathodes function at ∼1.2 V [11].

To overcome the voltage limitations in aqueous battery systems, the development of new chemistries is essential. In this context, we propose aqueous manganese‐ion batteries (AMIBs) as a promising alternative [14, 15, 16, 17, 18, 19, 20, 21, 22, 23, 24, 25]. AMIBs offer several advantages, including a low standard reduction potential (−1.19 V vs. SHE) [26, 27], which enables a higher operating voltage compared to aqueous zinc‐ion batteries. Additionally, manganese is an abundant and cost‐effective resource, ensuring sustainability, and has a high theoretical gravimetric capacity of 976 mAh g^−1^. Our previous study confirmed the reversible operation of a manganese metal anode in aqueous electrolytes [16, 19]. Furthermore, Mn^2+^ ions possess a relatively large hydrated radius (4.38 Å) compared to other divalent cations such as Zn^2+^ (4.30 Å), Ca^2+^ (3.34 Å), and Mg^2+^ (4.28 Å) [28]. This enhances the desolvation kinetics prior to host structure intercalation, contributing to improved cycle stability and high rate capabilities in electrochemical cells. However, AMIBs face a critical challenge: the direct utilization of manganese metal as an anode is hindered by the hydrogen evolution reaction (HER) on the manganese electrode surface [19]. This limits the practical application of Mn^2+^‐based aqueous electrolytes in battery systems. Recent studies have further emphasized that stabilizing aqueous Mn‐based systems requires careful regulation of interfacial chemistry (e.g., suppressing parasitic reactions and modulating proton activity/electric double‐layer effects) [29, 30]. Moreover, only a few viable cathode materials have been reported to date, including vanadium oxide‐based materials [29], Prussian blue frameworks [25], and organic electrodes [24]. Meanwhile, advanced cathode design concepts, such as dual‐storage mechanisms, have also been proposed to expand the accessible capacity and reaction pathways of Mn‐based batteries [31].

In this study, we demonstrate, for the first time, the use of monoclinic KV_3_O_8_ as a cathode material for AMIBs in a MnCl_2_‐saturated aqueous electrolyte. KV_3_O_8_ is an alkali vanadate material with an interlayer spacing of 7.63 Å, which facilitates efficient charge storage. Electrochemical performance was evaluated using cyclic voltammetry (CV) and galvanostatic charge–discharge testing. The material exhibited a redox potential of −0.05 V, corresponding to 1.14 V vs. Mn/Mn^2+^. Additionally, it demonstrated excellent cycling stability, retaining 88.0% of its initial capacity after 3600 cycles. Various spectroscopic techniques and X‐ray diffraction (XRD) analysis were employed to elucidate the charge storage mechanism of KV_3_O_8_. Furthermore, density functional theory (DFT) calculations and first‐principles molecular dynamics (FPMD) simulations were employed to elucidate the mechanism underlying the energy storage process involving manganese and protons. Finally, the Mn metal/KV_3_O_8_ cell exhibited a higher operating voltage compared to zinc‐based battery systems, underscoring the potential of AMIBs as a next‐generation energy storage technology. These findings provide new insights into the development of AMIBs and establish monoclinic KV_3_O_8_ as a highly stable cathode material, paving the way for the commercialization of high‐performance AMIBs for grid‐scale energy storage and other advanced energy applications.

## 2 Results and Discussion

### 2.1 Material Synthesis and Characterization of KV_3_O_8_

**FIGURE 1:**
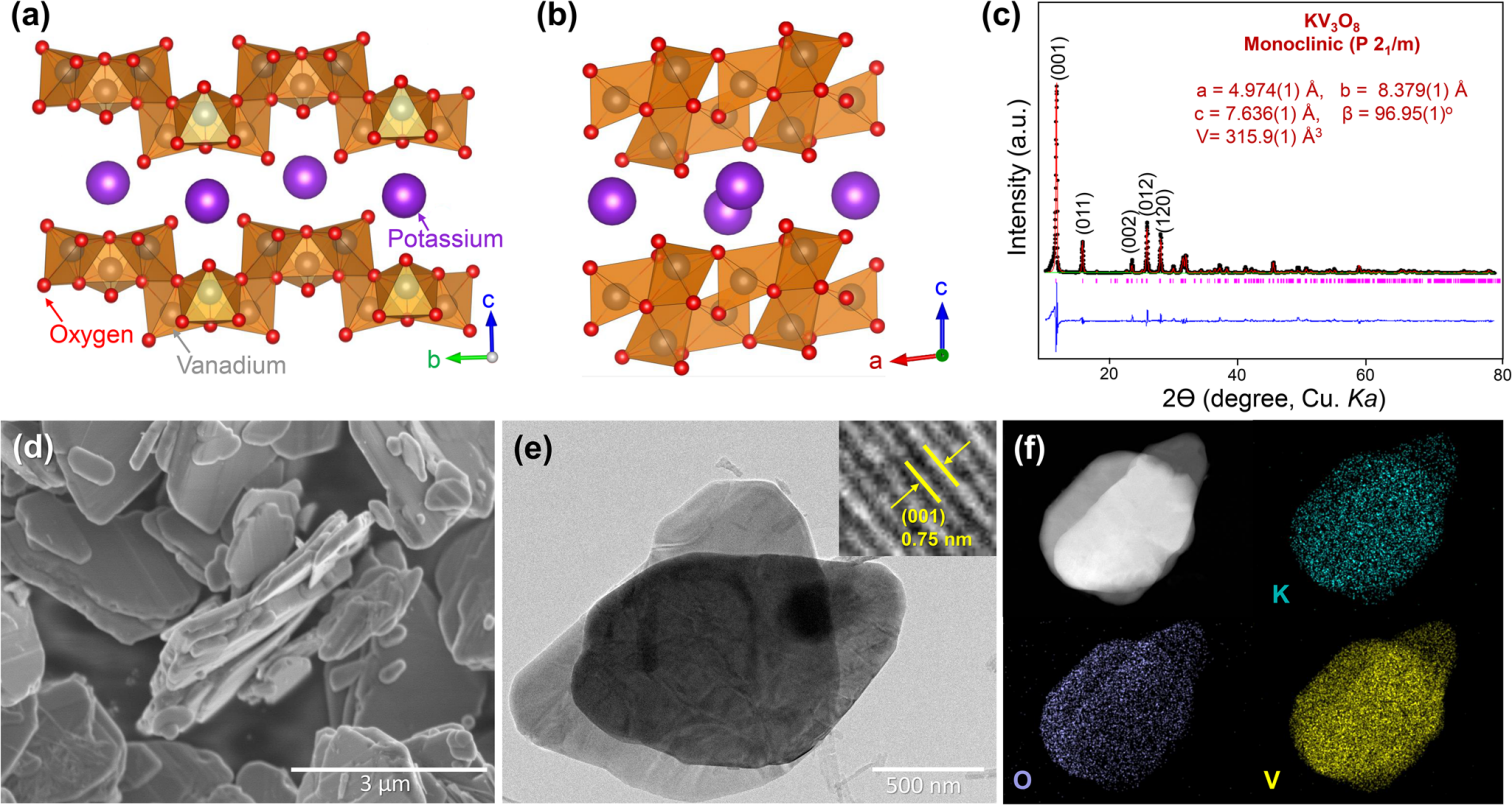
Crystal structure of KV_3_O_8_ viewed along the (a) bc‐plane and (b) ab‐plane. (c) Rietveld refinement profiles of KV_3_O_8_ from powder XRD data. (d) SEM image of the as‐prepared KV_3_O_8_ powder. (e) TEM image of the as‐prepared KV_3_O_8_ powder; (inset) high‐resolution lattice spacings. (f) TEM–EDX elemental mapping images of the KV_3_O_8_ powder for potassium (cyan), oxygen (purple), and vanadium (yellow).

Figure 1a, b depict the crystallographic structure of KV_3_O_8_ viewed along the bc‐plane and ab‐plane, respectively. KV_3_O_8_ adopts a monoclinic (*P2_1_/m*) crystal structure, where K^+^ ions reside in the interlayer region of vanadium oxides, forming a layered framework. The framework consists of interconnected VO_6_ octahedra, with some vanadium atoms adopting a VO_5_ square‐pyramidal coordination. Along the b‐axis, the VO_5_ groups form chains, which are further linked along the c‐axis to establish the layered structure. We hypothesize that KV_3_O_8_ is a highly stable host structure owing to the intercalated K^+^ ions, which possibly act as structural pillars, preventing the collapse of the layered structure upon cation insertion.

The synthesized KV_3_O_8_ was characterized by XRD, scanning electron microscopy (SEM), and transmission electron microscopy (TEM) to investigate its structural and morphological properties. Figure 1c presents the powder XRD pattern of KV_3_O_8_, analyzed by the Rietveld refinement method using the GSAS software [32]. The analysis confirms a monoclinic *P2_1_/m* symmetry, with lattice parameters of *a* = 4.974(1) Å, *b* = 8.379(1) Å, *c* = 7.636(1) Å, β = 96.95(1)°, and a unit cell volume of 315.9(1) Å^3^. The diffraction peaks are characteristic of the layered structure, further supporting the crystallographic integrity of KV_3_O_8_. Detailed Rietveld refinement parameters are listed in Table S1 of the Supporting Information. Figure 1d and Figure S1 present the SEM images of KV_3_O_8_, revealing the presence of nanosheets in the micrometer range. The nanosheet‐like morphology is consistent with the layered nature of KV_3_O_8_, suggesting its potential for facile ion diffusion. TEM analysis (Figure 1e) further confirms the flaky or thin‐layered nanostructure of KV_3_O_8_, with individual sheets extending to several hundred nanometers. The inset in Figure 1e presents a high‐resolution TEM image, clearly resolving the atomic arrangement within the crystalline structure. The periodic linear patterns correspond to specific lattice planes, with the (001) interlayer spacing measured at 7.5 Å, which is in good agreement with our Rietveld refinement results. The corresponding selected‐area electron diffraction pattern confirms its polycrystalline nature, revealing the presence of multiple small crystallites within the sample (Figure S2). The elemental distribution within the KV_3_O_8_ material was investigated using TEM–EDX elemental mapping, as presented in Figure 1f and Figure S3. The mapping results indicate a homogeneous distribution of potassium, oxygen, and vanadium throughout the structure, confirming the uniformity of the material composition. Overall, these characterizations confirm the successful synthesis of pure‐phase KV_3_O_8_ without impurities.

### 2.2 Electrochemical Charge Storage Performance of KV_3_O_8_

To evaluate the electrochemical Mn^2+^/H^+^ storage performance of KV_3_O_8_ electrodes in AMIBs, a three‐electrode beaker‐type cell was employed. The direct use of manganese metal as the anode was avoided because of the occurrence of the HER in the aqueous electrolyte. This side reaction could generate byproducts that block the active sites of the manganese metal, thereby degrading the electrochemical activity of the anode. To eliminate these unwanted effects and accurately assess the intrinsic electrochemical properties of KV_3_O_8_, the three‐electrode setup was used, comprising a KV_3_O_8_ working electrode, an activated carbon counter electrode, a glass microfiber separator, and a saturated MnCl_2_ aqueous electrolyte.

**FIGURE 2:**
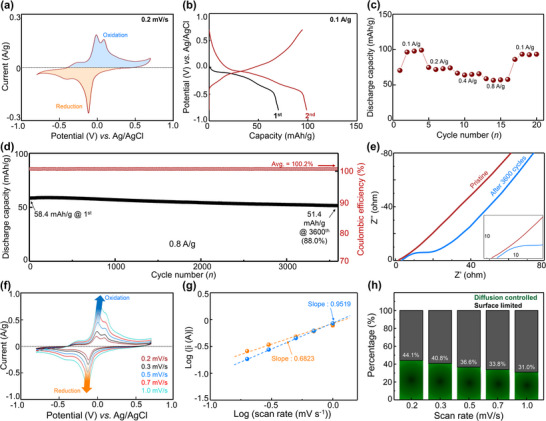
Electrochemical charge storage characteristics of KV_3_O_8_ electrode for AMIBs: (a) CV of KV_3_O_8_ electrode performed at 0.2 mV s^−1^, (b) GDC profiles of KV_3_O_8_ electrode at a current density of 0.1 A g^−1^ for the first (black) and second (red) cycles, (c) rate capability of the KV_3_O_8_ electrode under different current densities ranging from 0.1 to 0.8 A g^−1^, (d) long‐term cycling performance of the KV_3_O_8_ electrode over 3600 cycles at a current density of 0.8 A g^−1^, (e) Nyquist plots of KV_3_O_8_ electrodes in their pristine state and after 3600 cycles, (f) CV curves of the KV_3_O_8_ electrodes at various scan rates ranging from 0.2 to 1.0 mV s^−1^, (g) b‐values derived from the correlation between the cathodic peak current and scan rate, and (h) contribution ratio of capacitive and ion intercalation reactions in KV_3_O_8_.

The CV curves of the KV_3_O_8_ electrodes were recorded at a scan rate of 0.2 mV s^−1^ within a potential window ranging from –0.8 to 0.7 V (vs. Ag/AgCl) (Figure 2a). During the charging process, two distinct oxidation peaks were observed around 0.0 and 0.1 V, while a single, more pronounced reduction peak was identified around −0.1 V during the discharge process. This behavior suggests multi‐step intercalation/deintercalation of Mn^2+^/H^+^ within the layered KV_3_O_8_ structure. Galvanostatic discharge/charge (GDC) tests were performed within a potential range from −0.8 to 0.7 V at a current density of 0.1 A g^−1^. The first discharge curve and second charge/discharge cycle profiles are shown in Figure 2b. In the first cycle, the KV_3_O_8_ electrode exhibited a discharge capacity of 70.4 mAh g^−1^, while in the second cycle, the discharge capacity increased to 99.1 mAh g^−1^. To further investigate the electrochemical properties of the KV_3_O_8_ electrode, its rate capability was evaluated under various current densities (Figure 2c). The reversible discharge capacities at current densities of 0.1, 0.2, 0.4, and 0.8 A g^−1^ were 99.1, 74.7, 66.5, and 58.7 mAh g^−1^, respectively, demonstrating excellent rate performance. The corresponding GDC profiles are presented in Figure S4.

The cycling stability of the KV_3_O_8_ electrode was evaluated at a current density of 0.8 A g^−1^, as shown in Figure 2d. Although the initial discharge capacity was relatively low at 58.4 mAh g^−1^, the capacity gradually declined over 3600 cycles while maintaining an impressive capacity retention of 88.0% and a Coulombic efficiency of 100.2%. The detailed GDC curves for the first, 1000th, 2000th, and 3000th cycles are presented in Figure S5. Similar to previous observations (Figure 2d), the discharge capacity gradually decreased from the first cycle to the 3000th cycle. This capacity fading is attributed to structural degradation, as evidenced in Figure S6. Nevertheless, the electrode still maintains high capacity retention over 3600 cycles, indicating that KV_3_O_8_ evolves toward a disordered/amorphous vanadate phase without catastrophic structural failure. Importantly, the nearly unchanged Warburg slopes after long‐term cycling (Figure 2e) indicate that ion‐transport kinetics are largely preserved, suggesting that proton diffusion remains efficient even though the long‐range crystallinity of the host structure is substantially reduced.

To further investigate the electrochemical behavior of the KV_3_O_8_ cathode, electrochemical impedance spectroscopy (EIS) was performed to analyze resistance changes during cycling at a current density of 0.8 A g^−1^. As shown in Figure 2e, the small semicircle in the high‐frequency region corresponds to the charge transfer resistance (R_ct_) at the electrode/electrolyte interface, while the linear part in the low‐frequency region is associated with the Warburg impedance, reflecting cation diffusion within the electrode. The intrinsic resistance of the cell wiring increased from 2.39 to 4.59 Ω after long‐term cycling. The initial R_ct_ value was 5.65 Ω, which increased to 23.1 Ω after 3600 cycles, as evident from the significant increase in the semicircle diameter. This increase suggests the formation of surface side products, such as Mn(OH)_2_, on the KV_3_O_8_ electrode. Nevertheless, the Warburg slopes remain nearly unchanged even after 3600 cycles, indicating that the ion diffusion and charge storage performance within the electrode structure are well maintained. To gain deeper insights into the contribution of capacitive and intercalation processes in the KV_3_O_8_ electrode, CV profiles were recorded at scan rates ranging from 0.2 to 1.0 mV s^−1^ (Figure 2f). The cation storage behavior was analyzed using the power‐law relationship. The calculated b‐values (Figure 2g), which indicate the dominance of capacitive versus diffusion‐controlled processes, were 0.6823 for the reduction process and 0.9519 for the oxidation process. These results suggest that the capacity of KV_3_O_8_ is primarily governed by surface capacitive reactions, with a minor contribution from diffusion‐controlled intercalation. Quantitatively, approximately 44.1% of the cation storage is attributed to intercalation, while the remaining 55.9% is associated with surface‐limited behavior (Figure 2h). Furthermore, increasing the scan rate led to a noticeable increase in the capacitive contribution (Figure S7).

### 2.3 Charge Storage Mechanism of KV_3_O_8_

**FIGURE 3:**
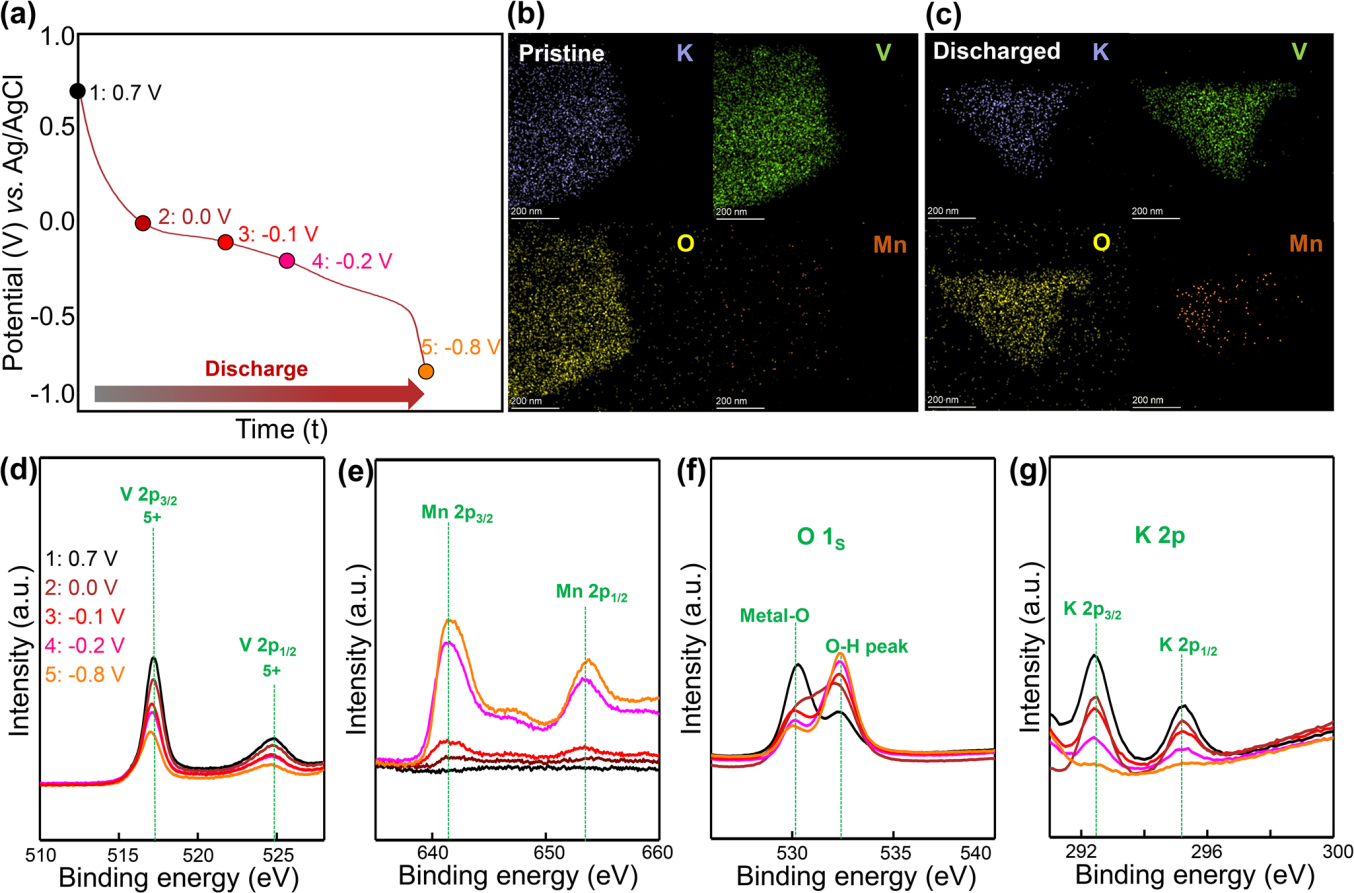
(a) Discharge profile of KV_3_O_8_ from the pristine state (0.7 V vs. Ag/AgCl) to the fully discharged state (−0.8 V vs. Ag/AgCl). TEM–EDX elemental mapping of KV_3_O_8_ in the (b) pristine (0.7 V vs. Ag/AgCl) and (c) fully discharged (−0.8 V vs. Ag/AgCl) states. XPS spectra of KV_3_O_8_ at different discharge states, showing the evolution of (d) V 2p, (e) Mn 2p, (f) O 1s, and (g) K 2p core levels.

To investigate the electrochemical charge storage mechanism and structural evolution of the KV_3_O_8_ cathode during the initial discharge cycle, TEM–EDX elemental mapping and XPS analyses were conducted at five specific potential states (Figure 3). As illustrated in the discharge profile (Figure 3a), measurements were taken at five distinct voltage points, ranging from the pristine state (0.7 V vs. Ag/AgCl) to the fully discharged state (−0.8 V vs. Ag/AgCl), including intermediate potentials of 0.0, −0.1, and −0.2 V. The TEM–EDX elemental mapping images of the KV_3_O_8_ electrode (Figure 3b, c) show no detectable manganese signal in the pristine state, while a small manganese signal appears in the fully discharged state. The corresponding EDX spectra (Figures S8 and S9) confirm this observation. These findings suggest that the Mn^2+^ ions from the aqueous MnCl_2_ electrolyte participate minimally in the charge storage mechanism, and instead, proton intercalation likely plays a more dominant role. Consistently, the recharged electrode does not show a meaningful decrease in the manganese signal compared to the fully discharged state (Figure S10).

**FIGURE 4:**
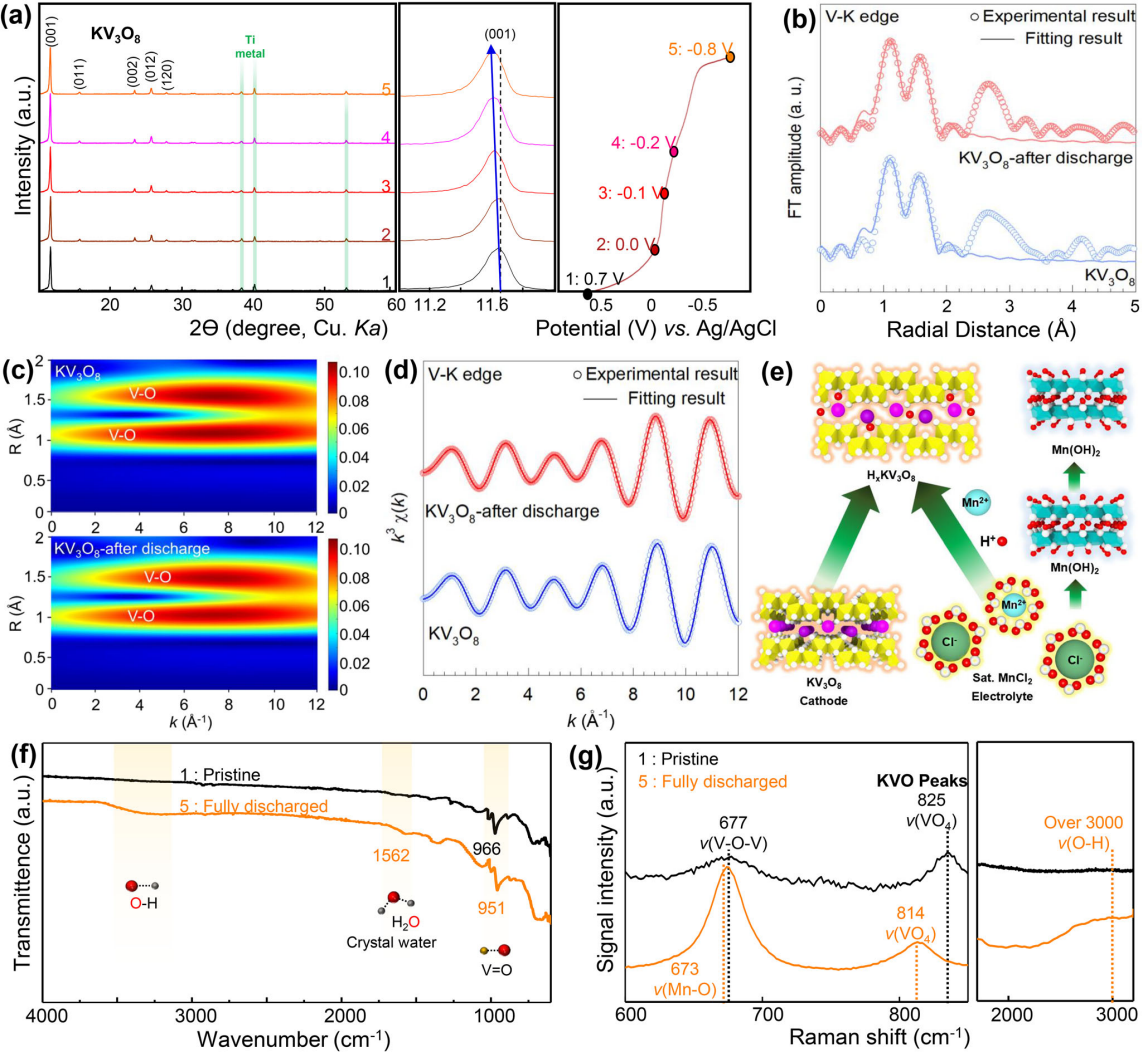
(a) Ex situ XRD patterns obtained at specific voltage points during the discharge process. (b) Ex situ V K‐edge FT‐EXAFS fitting data, (c) WT contour plots, and (d) Fourier‐filtered EXAFS spectra of KV_3_O_8_ before and after the discharging process. (e) Reaction mechanism of KV_3_O_8_ in saturated aqueous MnCl_2_ electrolyte. (f) FTIR and (g) Raman spectra of the KV_3_O_8_ electrode in pristine and fully discharged states.

To further confirm proton involvement, XPS analyses of V 2p, Mn 2p, O 1s, and K 2p regions were performed (Figure 3d–g). As shown in Figure 3d, the V^5+^ (2p_3/2_, 2p_1/2_) peaks decrease in intensity and shift to lower binding energies during discharge, indicating vanadium reduction (V^5+^ to V^4+^) due to cation insertion [33]. The separation of V^4+^ and V^5+^ components is further hindered by the discharge‐induced formation of a surface Mn(OH)_2_ layer, which significantly reduces the vanadium signal intensity. Under these conditions, the vanadium valence evolution is evaluated based on correlated XPS binding energy shifts and intensity trends, and further supported by V K‐edge EXAFS, Raman, and FTIR analyses (Figure 4). Meanwhile, on recharging, the V 2p spectra exhibit a clear recovery trend, where the V^5+^ peak intensity increases and the overall peak position shifts back toward higher binding energies, indicating vanadium re‐oxidation associated with cation extraction (Figure S11). While EDX did not detect significant manganese signals, XPS analysis revealed an increase in Mn 2p_3/2_ and 2p_1/2_ peak intensities during discharge (Figure 3e), suggesting a possible involvement of manganese. However, this manganese signal likely appears because of the formation of Mn(OH)_2_ on the electrode surface, either as a result of proton intercalation or side reactions, rather than direct manganese intercalation into the KV_3_O_8_ structure [18]. To support this hypothesis, the O 1s XPS spectra were examined (Figure 3f). The spectra reveal a noticeable increase in the O–H peak at 532.2 eV during discharge, confirming proton storage and the formation of Mn(OH)_2_ on the electrode surface. Additionally, the V─O bond peak at 530.1 eV decreases in intensity, consistent with the surface formation of Mn(OH)_2_ [18]. Furthermore, as shown in Figure 3g, the potassium signal also diminishes progressively with discharge, becoming nearly undetectable at the fully discharged state. This attenuation is likely due to the Mn(OH)_2_ layer covering the electrode surface. After re‐charged states, the Mn 2p, O 1s, and K 2p signals exhibit only partial recovery (Figure S11), which is consistent with the formation of a largely irreversible and electrically insulating Mn(OH)_2_ layer during discharge, thereby limiting the full recovery of these surface‐sensitive signals under the present ex situ conditions. The surface deposition of manganese ions after the discharge process was further confirmed by Mn K‐edge X‐ray absorption near‐edge structure (XANES) analysis for the fully discharged KV_3_O_8_ material, showing no distinct Mn K‐edge signal. This is in stark contrast to the observation of a notable Mn 2p XPS signal. Considering the bulk sensitivity of XANES and the surface sensitivity of XPS [34], the observed discrepancy between Mn K‐edge XANES and Mn 2p XPS data provides convincing evidence for the predominant surface deposition of Mn ions and the negligible intercalation of Mn ions into the interlayer space of the bulk KV_3_O_8_ lattice. In contrast to Mn K‐edge XANES data, ex situ V K‐edge XANES spectra of KV_3_O_8_ materials exhibit characteristic spectral features of V^5+^ species, including an intense pre‐edge peak P related to dipole‐forbidden 1s → 3d transition (Figure S12) [35]. The observation of intense pre‐edge peak P for pristine KV_3_O_8_ underscores the stabilization of vanadium ions in non‐centrosymmetric square‐pyramidal geometry and highly distorted octahedral geometry [35]. Before and after the discharge process, the overall spectral features of KV_3_O_8_ are well‐maintained, indicating the retention of the layered vanadium oxide framework.

Structural evolution of the KV_3_O_8_ electrode during the initial discharge cycle was investigated using ex situ XRD analysis, as shown in Figure 4a. Five prominent diffraction peaks corresponding to the (001), (011), (002), (012), and (120) planes were consistently observed throughout the discharge process, indicating that the layered structure of KV_3_O_8_ remained intact without any significant structural collapse during the first discharge. No additional diffraction peaks were observed compared to those in the pristine state, confirming the structural integrity of the KV_3_O_8_ framework. However, a distinct shift of the (001) peak toward a lower angle (from 11.65° to 11.60°) was detected. As the (001) plane is perpendicular to the c‐axis and reflects the interlayer spacing, this shift suggests a slight increase in the interlayer distance, attributed to partial proton insertion during the discharge process. During the subsequent re‐charge process, the (001) peak shifts back toward a higher angle (Figure S13), indicating partial recovery of the interlayer spacing and supporting the structural reversibility of the KV_3_O_8_ host on proton extraction.

The maintenance of the KV_3_O_8_ lattice during the initial discharging process was further evidenced by ex situ V K‐edge extended X‐ray absorption fine structure (EXAFS) analysis (Figure S14). As presented in Figure 4b, pristine KV_3_O_8_ material displays typical Fourier transform (FT) spectral features including intense peaks at approximately 1.2, 1.6, and 2.7 Å, which correspond to the V─O, V─O, and V─V coordination shells, respectively. This FT spectrum is characteristic of the KV_3_O_8_ lattice, wherein vanadium ions are stabilized in two square‐pyramidal sites and one octahedral site. The overall FT features of KV_3_O_8_ are well‐maintained with slight depression of V─O‐related peaks after the discharge process, demonstrating the retention of the crystal lattice and the increase of structural disorder. The good structural stability of KV_3_O_8_ and cycling‐induced disordering were further confirmed by wavelet transform (WT) contour data showing the maintenance of overall spectral features with the slight depression in the intensity maxima of the V─O bonding pairs after the discharging process, see Figure 4c. The quantitative information about the evolution of local structure upon electrochemical cycling was acquired by EXAFS fitting analysis. Since the vanadium ions in KV_3_O_8_ lattice have highly distorted local structures (i.e., two square pyramidal and one octahedral geometries) with various V─O bond distances from 1.58 to 2.26 Å, curve fitting analysis was conducted based on averaged structural model with two categories of V−O bond distances (e.g., ∼1.7 and ∼2.0 Å for average coordination numbers of 3 and 2.3, respectively), allowing to reproduce the observed two FT features of present materials, see Figure 4b, d. As listed in Table S2, the least squares curve fitting analysis reveals that the discharging process leads to the minute elongation of average V─O bond distances, supporting the reduction of V valence state. In addition, the V─O coordination shells exhibit slight decreases in the coordination number and increases in the Debye−Waller factor after the discharging process, substantiating the discharge‐driven disordering of the KV_3_O_8_ electrode.

The proposed reaction mechanism involving proton insertion and the formation of Mn(OH)_2_ is schematically illustrated in Figure 4e. In the saturated MnCl_2_ aqueous electrolyte, H^+^ ions intercalate into the KV_3_O_8_ structure, forming H_x_KV_3_O_8_. Simultaneously, the consumption of H^+^ ions increases the local concentration of OH^−^, promoting the precipitation of Mn(OH)_2_ on the electrode surface. To further support the proposed mechanism, FTIR (Figure 4f) and Raman spectroscopy (Figure 4g) analyses were conducted. In the FTIR spectrum (Figure 4f), a new peak emerges at approximately 3500 cm^−1^ in the fully discharged state, which is absent in the pristine state. This peak corresponds to the O─H stretching vibration, indicating the formation of Mn(OH)_2_ and indirectly confirming that proton insertion occurs more readily than manganese insertion. Additionally, a peak at 1562 cm^−1^ associated with crystal water appears, suggesting that water molecules may also be partially inserted into the structure during discharge. Moreover, after discharge (corresponding to the reduction of V^5+^ to V^4+^), the binding force between vanadium and oxygen decreases, as evidenced by the shift in the V═O stretching peak from 966 cm^−1^ in the pristine state to 951 cm^−1^ in the discharged state [36].

Figure 4g presents the Raman spectra, highlighting the shifts in the vibrational modes of V–O–V (677 cm^−1^) and VO_4_ (825 cm^−1^) as the electrode transforms from the pristine to the fully discharged state [37]. The VO_4_ peak shifts to 814 cm^−1^, consistent with vanadium reduction during discharge. This observation is consistent with the XPS and FTIR analyses, further confirming the valence change of vanadium. Additionally, the appearance of peaks corresponding to Mn─O vibrations at 673 cm^−1^ and O─H stretching vibrations above 3000 cm^−1^ in the fully discharged state suggests both proton insertion into the KV_3_O_8_ structure and the formation of Mn(OH)_2_ on the electrode surface. These findings imply that despite using a manganese‐based electrolyte, protons predominantly participate in the charge storage mechanism within the KV_3_O_8_ structure. This mechanism was further validated when a full cell was assembled using a manganese metal anode and a KV_3_O_8_ cathode, confirming that the device operates as a hybrid‐type battery.

**FIGURE 5:**
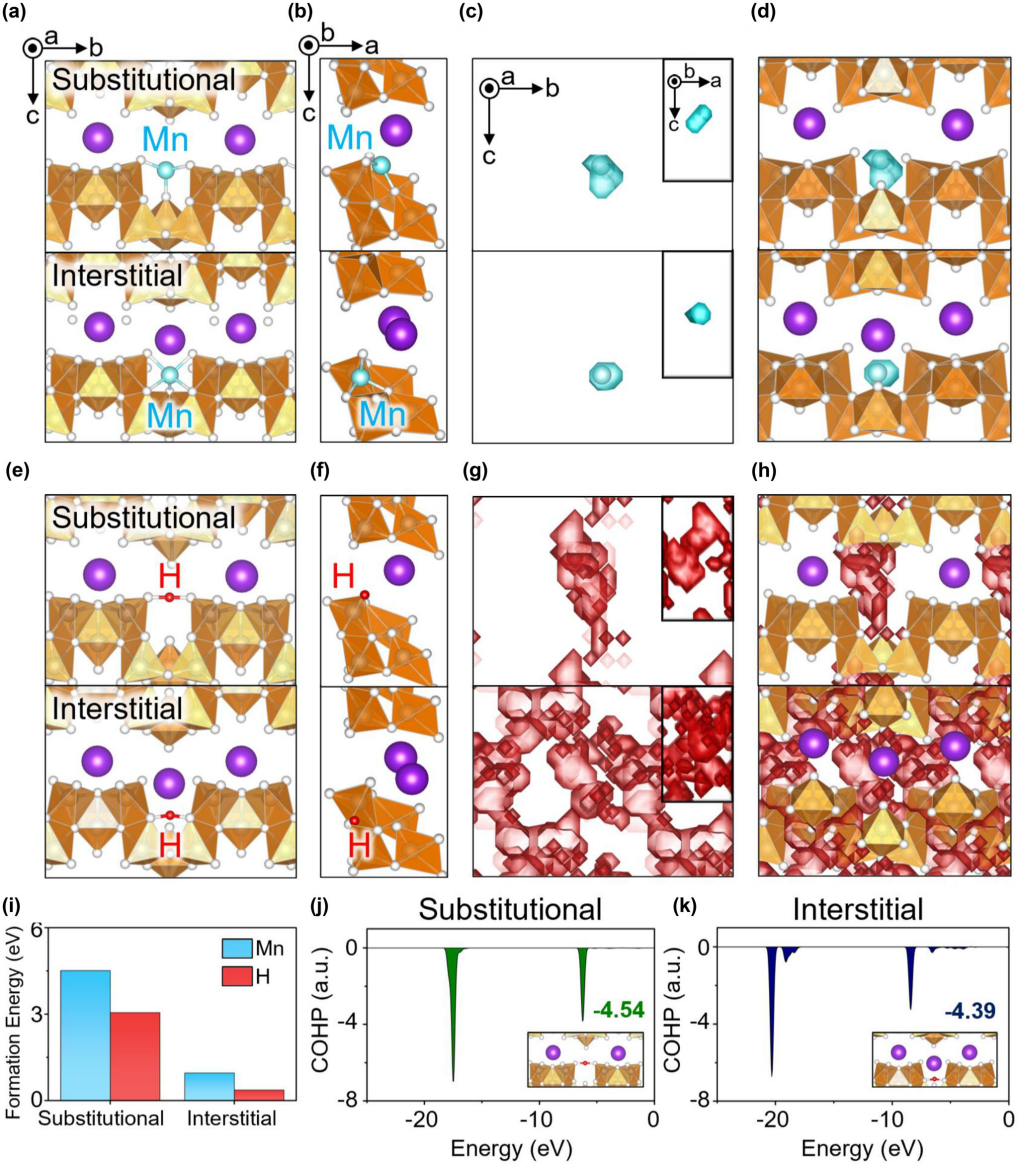
Atomic configurations of manganese at substitutional and interstitial sites in KV_3_O_8_ projected onto the (a) b‐c plane and (b) a‐c plane. (c) Diffusion pathways of manganese within the lattice are projected onto the b‐c plane, with the inset showing the projection onto the a‐c plane. (d) Diffusion pathways projected onto the crystal structures. Atomic configurations of a proton at substitutional and interstitial sites in KV_3_O_8_ projected onto the (e) b‐c plane and (f) a‐c plane. (g) Diffusion pathways of proton within the lattice projected onto the b‐c plane, with the inset showing the projection onto the a‐c plane. (h) Diffusion pathways projected onto the crystal structures. (i) Formation energies for the storage of manganese and proton at substitutional and interstitial sites. COHP analysis of O−H pairs with the proton positioned at (j) substitutional and (k) interstitial sites.

To gain deeper insights into the charge storage mechanism from an atomic‐scale perspective, DFT calculations and FPMD simulations were performed (Figure 5). Figure 5a shows two structural models projected onto the b‐c plane representing i) manganese substituting for potassium and ii) manganese occupying an interstitial site, both optimized after atomic relaxation. While potassium ions have a large ionic radius of ∼1.59 Å for 10‐coordination within the VO_x_ (VO_5_ and VO_6_) clusters, manganese ions have an ionic radius of less than ∼1 Å. When manganese substitutes for potassium, the optimized manganese positions may be located in the interstitial trench along the VO_x_ clusters, rather than directly at the K site, owing to size disparity. Therefore, we also considered the scenario in which manganese could occupy an interstitial site without substituting for potassium. Figure 5b shows the equivalent models projected onto the a‐c plane, suggesting that there is sufficient space in the trench to accommodate the manganese ion without inducing structural frustration.

To explore the possible manganese diffusion pathways within the lattice, FPMD simulations were performed for 200 ps (Figure 5c). In both structural models, the diffusion pathways of manganese were confined to regions near the initial position. Figure 5d displays the diffusion pathways in conjunction with the crystal structures. Figure 5e shows the structural models for the proton, and Figure 5f displays the side views, suggesting that the proton also prefers a position near the interstitial trench. Figure 5g visualizes the diffusion pathways of the proton. Proton diffusion within the oxide system is proposed to occur via a hopping process, which involves hydroxide rotation followed by transfer to an adjacent oxygen ion [38, 39]. When a proton substitutes for K, it exhibits dynamic diffusion along the c‐axis. This can be attributed to the undercoordinated oxygen atoms resulting from the absence of K, which attract the proton and facilitate its diffusion. Interestingly, the proton at the interstitial site exhibited a 3D diffusion network throughout the KV_3_O_8_ structure, indicating that an unbiased and isotropic route for proton diffusion is available within an intact structural framework (Figure 5h). These calculation results therefore describe the intrinsic proton‐storage preference and diffusion characteristics of KV_3_O_8_ in its crystalline state. On long‐term electrochemical cycling, KV_3_O_8_ undergoes pronounced amorphization, as evidenced by ex situ XRD (Figure S6). In this regime, while the atomistic diffusion pathways identified from the crystalline model are not expected to be directly preserved, the nearly unchanged Warburg impedance (Figure 2e) after 3600 cycles indicates that proton transport remains kinetically favorable, suggesting that the fundamental proton‐dominant charge‐storage behavior persists even as long‐range structural order is lost. To compare the thermodynamic stability upon incorporation of manganese or hydrogen into KV_3_O_8_, formation energies were calculated (Figure 5i). For the structure models representing the substitution for potassium and intercalation at the interstitial trench, proton incorporation had a lower energy cost than manganese incorporation. To gain a fundamental understanding of the extended diffusion path in an intact atomic environment, the bonding strength between the proton and oxygen ions was evaluated by calculating the crystal orbital Hamiltonian population (COHP). Figure 5j, k show the COHP diagram for the proton‐oxygen pairs when a proton substitutes for potassium and when the proton is incorporated into the interstitial site, respectively. The integrated COHP values, with more negative values indicating stronger bonds, were −4.54 and −4.39 for the substitutional and interstitial models, respectively. The undercoordinated oxygen ions resulting from potassium substitution attract the proton more strongly, creating a preferential diffusion pathway along the c‐axis, while those in the intact structural framework provide a more extensive diffusion network. These computational results reveal the elemental diffusion process within KV_3_O_8_ and support the experimental findings that the proton is more prone to intercalation compared to manganese.

### 2.4 Comparison of Manganese and Zinc Cell Using KV_3_O_8_ Cathode

**FIGURE 6:**
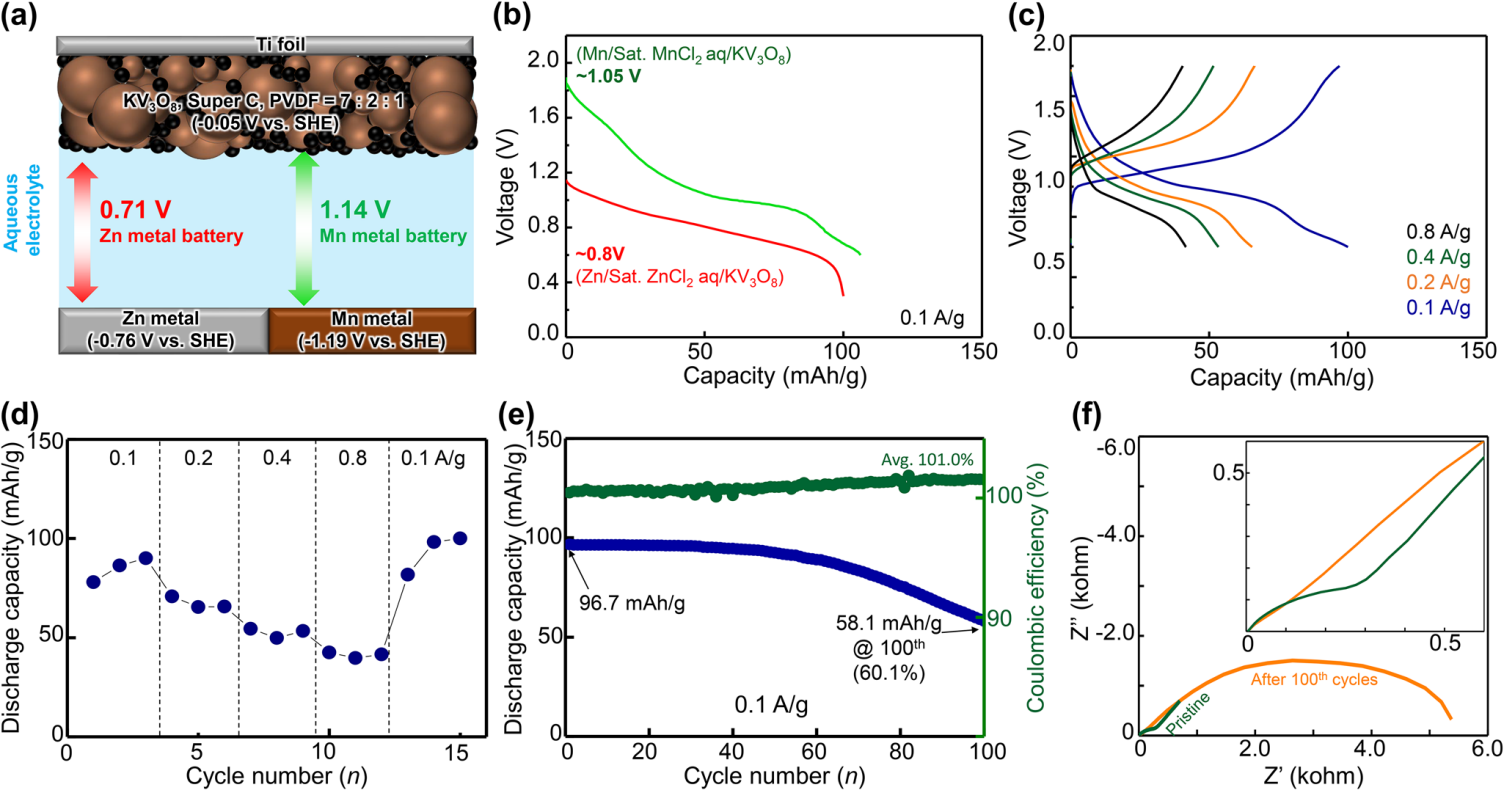
(a) Schematic illustration of the theoretical voltage differences between zinc metal and manganese metal batteries using a KV_3_O_8_ cathode in an aqueous electrolyte system. (b) Discharge voltage profiles of Zn//KV_3_O_8_ and Mn//KV_3_O_8_ cells at 0.1 A g^−1^. (c) GDC profiles and (d) rate capability of Mn//KV_3_O_8_ cells at different current densities (0.1, 0.2, 0.4, and 0.8 A g^−1^). (e) Long‐term cycling at 0.1 A g^−1^. (f) Nyquist plots of Mn//KV_3_O_8_ cells before and after 100 cycles.

Electrochemical measurements were conducted on manganese and zinc metal cells to evaluate the feasibility of manganese metal as an anode in aqueous systems and to compare its performance with zinc metal (Figure 6). As shown in Figure 6a, manganese metal exhibits a standard reduction potential of −1.19 V vs. SHE, which is more negative than that of zinc (−0.76 V vs. SHE), indicating the potential for a higher operating voltage when paired with KV_3_O_8_ cathodes. To experimentally validate this, discharge voltage profiles at 0.1 A g^−1^ were obtained (Figure 6b). The manganese metal cell in saturated MnCl_2_ electrolyte exhibited an operating voltage of ∼1.05 V, approximately 0.25 V higher than the zinc metal cell (∼0.8 V). Additionally, CV profiles for both cells (Figure S15) revealed more pronounced redox peaks for the manganese metal cell, suggesting that the MnCl_2_ electrolyte facilitated faster electron and ion transport compared to the ZnCl_2_ electrolyte. The GDC profiles of the manganese metal cell at various current densities are presented in Figure 6c, d. At 0.1 A g^−1^, the manganese metal cell delivered a discharge capacity comparable to that of an activated carbon anode system (Figure 2c). However, with increasing current density, a significant decline in capacity was observed: 65.5 mAh g^−1^ at 0.2 A g^−1^, 53.3 mAh g^−1^ at 0.4 A g^−1^, and 41.6 mAh g^−1^ at 0.8 A g^−1^, indicative of poor rate capability. This deterioration is attributed to the formation of a passivation layer on the manganese anode surface, primarily due to HER byproducts, which hinder charge transfer. Despite these challenges, the cycling stability of the manganese metal cell was evaluated over 100 cycles at 0.1 A g^−1^ (Figure 6e). The cell maintained relatively stable capacity retention for the first 50 cycles. However, after 100 cycles, the discharge capacity declined from an initial 96.7 to 58.1 mAh g^−1^, corresponding to 60.1% capacity retention. The corresponding GDC curves are shown in Figure S16. To elucidate the degradation mechanism, EIS was performed (Figure 6f). The internal resistance (R_s_) of the KV_3_O_8_ cathode increased from 3.21 to 9.21 Ω after 100 cycles. Notably, R_ct_ increased drastically from 458.5 to 5503 Ω, a significantly larger increase compared to that observed in Figure 2e. This indicates that the main source of resistance is the passivation layer on the manganese anode rather than the cathode–electrolyte interface layer.

Overall, this study demonstrates that manganese‐based batteries offer a higher energy density compared to conventional zinc‐based aqueous batteries. However, manganese‐based battery technology remains in its early stages, with critical challenges, particularly those related to the HER, which are yet to be addressed. Mitigation strategies, such as the optimization of electrolyte pH or the application of artificial protective layers on the manganese metal anode, could be effective in suppressing the HER and improving manganese battery performance.

## 3 Conclusion

In summary, this study is the first to introduce monoclinic KV_3_O_8_ as a cathode material exhibiting unprecedented electrochemical properties in AMIBs. KV_3_O_8_ with a layered structure was successfully synthesized at room temperature via a simple water bath method, demonstrating excellent structural stability and outstanding electrochemical performance. Our results confirm the reversible intercalation/deintercalation of protons within the KV_3_O_8_ framework. Notably, the KV_3_O_8_ cathode delivers exceptional cycling stability, retaining 88.0% of its initial capacity after 3600 cycles at 0.8 A g^−1^. Through a combination of XPS, ex situ XRD, XANES/EXAFS, FTIR spectroscopy, and Raman spectroscopy, the charge storage mechanism, including proton intercalation and Mn(OH)_2_ formation, was comprehensively elucidated. DFT calculations and FPMD simulations suggest a facile diffusion pathway for protons within the KV_3_O_8_ structural frame, indicating that protons are more readily incorporated into the lattice than manganese. Furthermore, the manganese metal cell in the saturated MnCl_2_ electrolyte exhibited a higher operating voltage (∼1.05 V) compared to its zinc counterpart (∼0.8 V), highlighting the potential of manganese‐based systems for high‐energy applications. This work not only offers valuable insights into the reaction mechanisms and byproduct formation in manganese‐ion batteries but also presents KV_3_O_8_ as a novel cathode material in manganese‐based hybrid batteries. Overall, these findings provide a foundation for the development of next‐generation, high‐energy, and stable cathode materials for future aqueous battery technologies.

## Conflicts of Interest

The authors declare no conflict of interest.

## Supporting information




**Supporting File**: advs74524‐sup‐0001‐SuppMat.pdf.

## Data Availability

The data that support the findings of this study are available from the corresponding author upon reasonable request.

## References

[1] M. Armand and J.‐M. Tarascon , “Building Better Batteries,” Nature 451 (2008): 652–657, 10.1038/451652a. 18256660

[2] K. Liu , Y. Liu , D. Lin , A. Pei , and Y. Cui , “Materials for Lithium‐Ion Battery Safety,” Science Advances 4 (2018): aas9820, 10.1126/sciadv.aas9820. PMC6014713 29942858

[3] Y. Chen , Y. Kang , Y. Zhao , et al., “A Review of Lithium‐ion Battery Safety Concerns: the Issues, Strategies, and Testing Standards,” Journal of Energy Chemistry 59 (2021): 83–99, 10.1016/j.jechem.2020.10.017.

[4] J. Wen , Y. Yu , and C. Chen , “A Review on Lithium‐Ion Batteries Safety Issues: Existing Problems and Possible Solutions,” Materials Express 2 (2012): 197–212, 10.1166/mex.2012.1075.

[5] Y. Liang and Y. Yao , “Designing Modern Aqueous Batteries,” Nature Reviews Materials 8 (2023): 109–122, 10.1038/s41578-022-00511-3.

[6] D. Chao , W. Zhou , F. Xie , et al., “Roadmap for Advanced Aqueous Batteries: From Design of Materials to Applications,” Science Advances 6 (2020): aba4098. 10.1126/sciadv.aba4098 PMC7244306 32494749

[7] S. Chen , Y. Kong , C. Tang , et al., “Doping Regulation Stabilizing δ‐MnO_2_ Cathode for High‐Performance Aqueous Aluminium‐Ion Batteries,” Small 20 (2024): 2312229, 10.1002/smll.202312229. 38488721

[8] Y. Xu , C. Li , W. Deng , et al., “Achieving High Performance in Aqueous Iron‐Ion Batteries Using Tunnel‐Like VO_2_ as a Cathode Material,” Chemical Communications 59 (2023): 8576–8579, 10.1039/D3CC02038J. 37340786

[9] C. Li , Y. Xu , W. Deng , et al., “Regulating Interlayer‐Spacing of Vanadium Phosphates for High‐Capacity and Long‐Life Aqueous Iron‐Ion Batteries,” Small 20 (2024): 2305766, 10.1002/smll.202305766. 37771178

[10] Y. Xu , X. Wu , S. K. Sandstrom , et al., “Fe‐Ion Bolted VOPO_4_ 2H_2_O as an Aqueous Fe‐Ion Battery Electrode,” Advanced Materials 33 (2021): 2105234, 10.1002/adma.202105234. 34623704

[11] C. Xu , B. Li , H. Du , and F. Kang , “Energetic Zinc Ion Chemistry: the Rechargeable Zinc Ion Battery,” Angewandte Chemie 124 (2012): 957–959, 10.1002/ange.201106307. 22170816

[12] M. S. Chae , R. Attias , B. Dlugatch , Y. Gofer , and D. Aurbach , “Multifold Electrochemical Protons and Zinc Ion Storage Behavior in Copper Vanadate Cathodes,” ACS Applied Energy Materials 4 (2021): 10197–10202, 10.1021/acsaem.1c02075.

[13] M. Wu , C. Shi , J. Yang , et al., “The LiV_3_O_8_ Superlattice Cathode with Optimized Zinc Ion Insertion Chemistry for High Mass‐Loading Aqueous Zinc‐Ion Batteries,” Advanced Materials 36 (2024): 2310434, 10.1002/adma.202310434. 38439064

[14] S. Dong , Z. Xu , Z. Cao , et al., “Aqueous “Rocking‐chair” Mn‐ion Battery Based on an Industrial Pigment Anode,” Chemical Engineering Journal 501 (2024): 157774, 10.1016/j.cej.2024.157774.

[15] Z. Fan , Z. Hou , W. Lu , et al., “Combination Displacement/Intercalation Reaction of Ag 0.11 V_2_O_5_ Cathode Realizes Efficient Manganese Ion Storage Properties,” Small 21 (2025): 2406501, 10.1002/smll.202406501. 39449541

[16] J. Pyun , H. Lee , H. Lee , et al., “The Charge Storage Mechanism and Durable Operation in Olivine–Lithium–Iron–Phosphate for Mn‐Based Hybrid Batteries,” Advanced Science 12 (2025): 2502866, 10.1002/advs.202502866. 40091612 PMC12097060

[17] Z. Cheng , Q. Dong , G. Pu , J. Song , W. Zhong , and J. Wang , “A Durable and High‐Voltage Mn–Graphite Dual‐Ion Battery Using Mn‐Based Hybrid Electrolytes,” Small 20 (2024): 2400389, 10.1002/smll.202400389. 38287734

[18] H. Lee , H. Lee , J. Pyun , S. T. Hong , and M. S. Chae , “Monoclinic Silver Vanadate (Ag0. 33V_2_O_5_) as a High‐Capacity Stable Cathode Material for Aqueous Manganese Batteries,” Advancement of Science 11 (2024): 2406642. 10.1002/advs.202406642 PMC11496989 39135537

[19] H. Lee , A. Nimkar , H. Lee , et al., “New Mn Electrochemistry for Rechargeable Aqueous Batteries: Promising Directions Based on Preliminary Results,” Energy and Environmental Materials 8 (2025): 12823.

[20] S. Bi , Y. Zhang , S. Deng , Z. Tie , and Z. Niu , “Proton‐Assisted Aqueous Manganese‐Ion Battery Chemistry,” Angewandte Chemie International Edition 61 (2022): 202200809, 10.1002/anie.202200809. 35192232

[21] S. Bi , S. Wang , F. Yue , Z. Tie , and Z. Niu , “A Rechargeable Aqueous Manganese‐ion Battery Based on Intercalation Chemistry,” Nature communications 12 (2021): 6991. 10.1038/s41467-021-27313-5 PMC8632892 34848734

[22] Q. Yang , X. Qu , H. Cui , et al., “Rechargeable Aqueous Mn‐Metal Battery Enabled by Inorganic–Organic Interfaces,” Angewandte Chemie International Edition 61 (2022): 202206471, 10.1002/anie.202206471. 35652288

[23] D. Shen , X. Zheng , R. Luo , et al., “A Rechargeable, Non‐aqueous Manganese Metal Battery Enabled by Electrolyte Regulation,” Joule 8 (2024): 780–798.

[24] H. Lee , A. Nimkar , N. Shpigel , et al., “π‐Electron‐Assisted Charge Storage in Fused‐Ring Aromatic Carbonyl Electrodes for Aqueous Manganese‐Ion Batteries,” ACS Energy Letters 9 (2024): 5627–5634, 10.1021/acsenergylett.4c02418.

[25] A. Nimkar , M. S. Chae , S. Wee , et al., “What about Manganese? Toward Rocking Chair Aqueous Mn‐Ion Batteries,” ACS Energy Letters (2022): 4161–4167, 10.1021/acsenergylett.2c02242.

[26] M. Wang , Y. Meng , Y. Xu , et al., “Aqueous All‐manganese Batteries,” Energy & Environmental Science 16 (2023): 5284–5293, 10.1039/D3EE01679J.

[27] M. Wang , X. Zheng , X. Zhang , et al., “Opportunities of Aqueous Manganese‐Based Batteries with Deposition and Stripping Chemistry,” Advanced Energy Materials 11 (2021): 2002904.

[28] S. Fan , L. Jiang , Z. Jia , Y. Yang , and L. Hou , “Comparison of Adsorbents for Cesium and Strontium in Different Solutions,” Separations 10 (2023): 266.

[29] M. Li , C. Li , C. Zuo , et al., “Strategically Modulating Proton Activity and Electric Double Layer Adsorption for Innovative All‐Vanadium Aqueous Mn_2_+ /Proton Hybrid Batteries,” Advanced Materials 36 (2024): 2407233, 10.1002/adma.202407233. 39152942

[30] M. Wang , Y. Meng , Y. Xu , D. Shen , P. Tong , and W. Chen , “An Energetic Aqueous Mn Metal Anode,” ACS Energy Letters 9 (2024): 1381–1388, 10.1021/acsenergylett.4c00103.

[31] D. Shen , G. Zhao , T. Jiang , et al., “A High‐Capacity Manganese‐Metal Battery with Dual‐Storage Mechanism,” Angewandte Chemie International Edition 64 (2025): 202423921, 10.1002/anie.202423921. 39887528

[32] B. H. Toby , “EXPGUI , a Graphical User Interface for GSAS,” Journal of Applied Crystallography 34 (2001): 210–213, 10.1107/S0021889801002242.

[33] H. J. Kim , J. H. Jo , J. U. Choi , N. Voronina , and S.‐T. Myung , “KV_3_O_8_ With a Large Interlayer as a Viable Cathode Material for Zinc‐ion Batteries,” Journal of Power Sources 478 (2020): 229072, 10.1016/j.jpowsour.2020.229072.

[34] S.‐J. Hwang , D. H. Park , and J.‐H. Choy , “Influence of Host Lattice on the Chemical Bonding Nature of Guest Species in High‐T c Superconducting I− Bi_2_Sr1. 5‐x La X Ca_1.5_Cu_2_O_y_ Nanohybrids,” Journal of Physical Chemistry B 108 (2004): 12044–12048.

[35] J. M. Lee and S.‐J. Hwang , “Remarkable Influence of the Local Symmetry of Substituted 3d Metal Ion on Bifunctional Electrocatalyst Performance of α‐MnO_2_ Nanowire,” Journal of Solid State Chemistry 269 (2019): 354–360.

[36] B. Feng , D. Sun , H. Wang , S. Tan , and H. Zhang , “A Simple Method for the Synthesis of KV_3_O_8_0.4_2_H_2_O Nanorod and Its Lithium Insertion/Deinsertion Properties,” International Journal of Electrochemical Science 8 (2013): 1095–1102, 10.1016/S1452-3981(23)14083-1.

[37] R. Baddour‐Hadjean , L. Thanh Nguyen Huynh , D. Batyrbekuly , S. Bach , and J. P. Pereira‐Ramos , “Bilayered Potassium Vanadate K 0.5 V_2_O_5_ as Superior Cathode Material for Na‐Ion Batteries,” Chemsuschem 12 (2019): 5192–5198, 10.1002/cssc.201902093. 31595706

[38] D. Kim , I. Jeong , S. Ahn , et al., “On the Role of Bimetal‐Doped BaCoO 3−𝛿 Perovskites as Highly Active Oxygen Electrodes of Protonic Ceramic Electrochemical Cells,” Advanced Energy Materials 14 (2024): 2304059, 10.1002/aenm.202304059.

[39] N. Tsvetkov , D. Kim , I. Jeong , et al., “Advances in Materials and Interface Understanding in Protonic Ceramic Fuel Cells,” Advanced Materials Technologies 8 (2023): 2201075, 10.1002/admt.202201075.

