# Supplementary material for: A Proton‐Intercalation Pathway Realizes Long‐Life Manganese‐Ion Hybrid Batteries With Layered KV3O8
Source: Adv Sci (Weinh). 2026 Feb 23;13(25):e74524. doi: 10.1002/advs.74524 (PMC13137789; doi:10.1002/advs.74524)
Supplement: Supplementary file 1 — Supporting File: advs74524‐sup‐0001‐SuppMat.pdf. [file ADVS-13-e74524-s001.pdf]

Supporting Information

A Proton-Intercalation Pathway Realizes Long-Life Manganese-Ion Hybrid Batteries with Layered KV_3O_8

Sangki Lee, Hyungjin Lee, Jangwook Pyun, Hyeonjun Lee, Ki-Min Roh, Kang Taek Lee, Seung-Tae Hong, Xiaoyan Jin, Incheol Jeong*, Seong-Ju Hwang*, Munseok S. Chae**

Experimental Methods

Material Synthesis and Characterizations

Monoclinic KV_3O_8 was synthesized through a liquid–solid stirring process at room temperature. Initially, 3.0 g of V_2O_5 (≥99%, Sigma-Aldrich) and 2.0 M potassium chloride (≥99%, Sigma-Aldrich) were added to 100 mL of deionized water and stirred vigorously at room temperature for three days. During this period, the solution gradually changed to a reddish-brown color. The resulting product was washed thoroughly with deionized water and ethanol, followed by centrifugation to obtain KV_3O_8. The obtained material was then dried at 80 °C for 12 h to complete the synthesis.

The crystal structure of the synthesized material was analyzed by powder X-ray diffraction (XRD; Rigaku Mini-Flex 600) using Cu Kα radiation, covering a 2θ range of 10°–80°. Rietveld refinements were performed using the GSAS program.[1] Morphology was characterized by scanning electron microscopy (SEM; Hitachi SU-8020, Japan) and transmission electron microscopy (TEM; FEI, Themis Z). Additionally, elemental mapping was performed using energy-dispersive X-ray spectroscopy (EDX) to enable both qualitative and quantitative elemental analysis. The oxidation states of the each elements were examined by X-ray photoelectron spectroscopy (XPS, AXIS SUPRA, KRATOS Analytical Ltd.). The V K-edge and Mn K-edge X-ray absorption near edge structure (XANES)/extended X-ray absorption fine structure (EXAFS) data were recorded at beamline 10C of Pohang Accelerator Laboratory (PAL, Republic of Korea) to probe the impact of discharge process on the metal oxidation state and local crystal structure of KV_3O_8. Fourier transform infrared spectroscopy (FTIR, Perkin-Elmer/Spectrum 3) was performed in the wavenumber range of 650-4000 cm^{-1}, and Raman spectroscopy (JASCO/NRS-5100) was conducted over a Raman shift range of 200-4000 cm^{-1} to further identify the vibrational characteristics of the electrode materials.

Electrochemical Characterization

The cathode was prepared by mixing potassium vanadium bronze (KV_3O_8; cathode material), Super P (conductive additive), and polyvinylidene fluoride (PVDF, binder) in a weight ratio of 7:2:1. The mixture was dispersed in *N*-methyl-2-pyrrolidone (NMP) to form a slurry, which was homogenized using a Thinky mixer at 1500 rpm for 30 min. The slurry was then cast onto a 30 µm-thick titanium foil, dried at 70 °C for 12 h, and compressed using a heated roll press. The final cathode had an active area of 1.33 cm^2 and contained approximately 1.4 mg of active

material. Electrochemical performance was evaluated using a three-electrode beaker-type cell within a potential window from –0.8 to 0.7 V vs. Ag/AgCl. A saturated $MnCl_2$ solution served as the aqueous electrolyte, glass microfiber paper (Whatman) as the separator, and activated carbon as the counter electrode. To ensure uniform ion intercalation, the assembled cell was wet for 10 h prior to testing. Galvanostatic discharge/charge (GDC) cycling and cyclic voltammetry (CV) measurements were performed using a VMP-3e potentiostat (BioLogic Science Instruments SAS) within the same voltage window. Potentiostatic electrochemical impedance spectroscopy (EIS) was conducted with a 10 mV AC amplitude over a frequency range of 0.1 Hz–200 kHz.

Computational Details

Density functional theory (DFT) calculations and first-principles molecular dynamics (FPMD) simulations were performed using the Vienna Ab-initio Simulation Package.[2] The Perdew–Burke–Ernzerhof generalized gradient approximation was considered for the exchange-correlation functionals. A 24-atom supercell was modeled for KV_3O_8. Projector-augmented-wave potentials were used with valence configurations of $3p^6 4s^1$, $3p^6 3d^4 4s^1$, $2s^2 2p^4$, $3p^6 3d^6 4s^1$, and $1s^1$ for K, V, O, Mn, and H, respectively. Plane waves with a cutoff energy of 500 eV were employed, and a 2 × 1 × 1 k-point mesh within the Monkhorst–Pack scheme[3] was adopted. The cutoff energy and k-point sampling were determined based on total energy convergence with a criterion of 0.01 eV per atom. The electronic self-consistent iteration threshold was set to 10^{-6} eV/cell. The cell parameters and atomic positions were relaxed until the residual force was below 0.1 eV/Å. FPMD simulations were performed within the NVT ensemble at 600 K for 200 ps with a time step of 2 fs.

References

[1] B. H. Toby, *Appl. Cryst.* **2001**, 34, 210.

[2] G. Kresse, J. Furthmüller, *Phys. Rev. B* **1996**, 54, 11169.

[3] H. J. Monkhorst, J. D. Pack, *Phys. Rev. B* **1976**, 13, 5188.

Table S1. Crystallographic data and Rietveld refinement data for highly crystalline KV_3O_8 using XRD data of the powders: atomic coordinates; site occupancies; and isotropic displacement parameters.

Crystal system	Monoclinic
Space group	$P\ 2_1/m$
Lattice parameter	a = 4.974(1) Å, b = 8.379(1) Å, c = 7.636(1) Å, V= 315.9(1) Å^3

Atoms	x	y	z	Wyckoff	Occupancy	U_{iso}
K(1)	0.0465(3)	0.2500	0.9394(3)	2	1.000	0.005(1)
V(1)	0.4480(3)	0.5571(3)	0.6891(3)	4	1.000	0.005(1)
V(2)	0.0775(3)	0.2500	0.4262(3)	2	1.000	0.005(1)
O(1)	0.1255(3)	0.6060(3)	0.7362(3)	4	1.000	0.005(1)
O(2)	0.3161(3)	0.0888(3)	0.4952(3)	4	1.000	0.005(1)
O(3)	0.4096(3)	0.5649(3)	0.1717(3)	4	1.000	0.005(1)
O(4)	0.1179(3)	0.7500	0.4255(3)	2	1.000	0.005(1)
O(5)	0.4092(3)	0.2500	0.2470(3)	2	1.000	0.005(1)

* R_p = 0.214, R_{wp} = 0.285, R_{exp} = 0.140, $R(F^2)$ = 0.17139, χ^2 = 4.162

Table S2. Results of non-linear least-squares V K-edge EXAFS fitting analysis of KV_3O_8 before and after discharging process.

Material	Bonding pair	Coordination number	R(Å)	ΔE (eV)	σ^2 (Å^2)
KV_3O_8	V–O	3	1.566	–4.84	0.0035
	V–O	2.3	1.920	1.58	0.0022
KV_3O_8-after discharging process	V–O	2.9	1.574	–4.31	0.0040
	V–O	2.2	1.928	2.19	0.0024

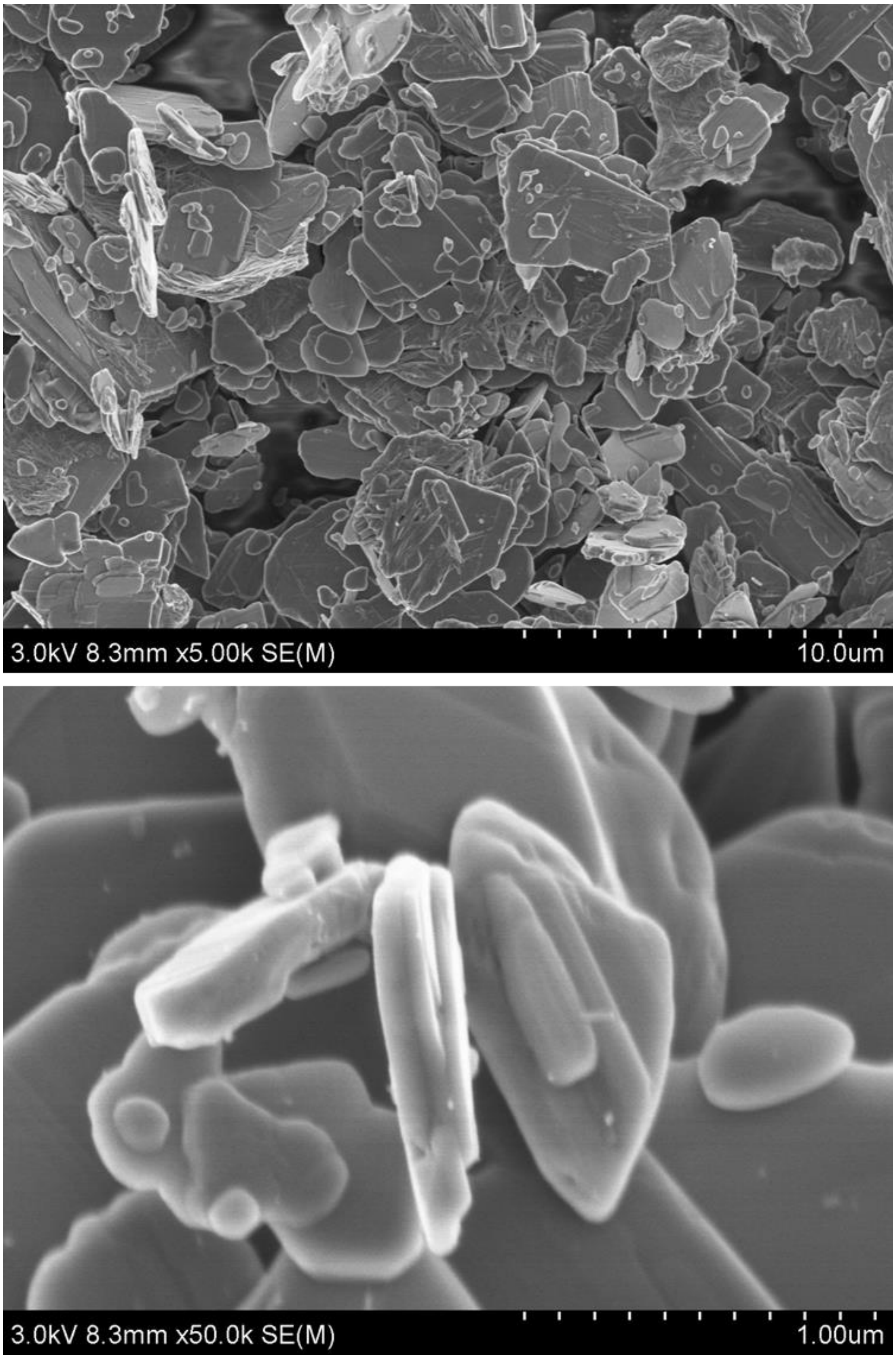

Figure S1. SEM images of the as-prepared KV_3O_8 powder.

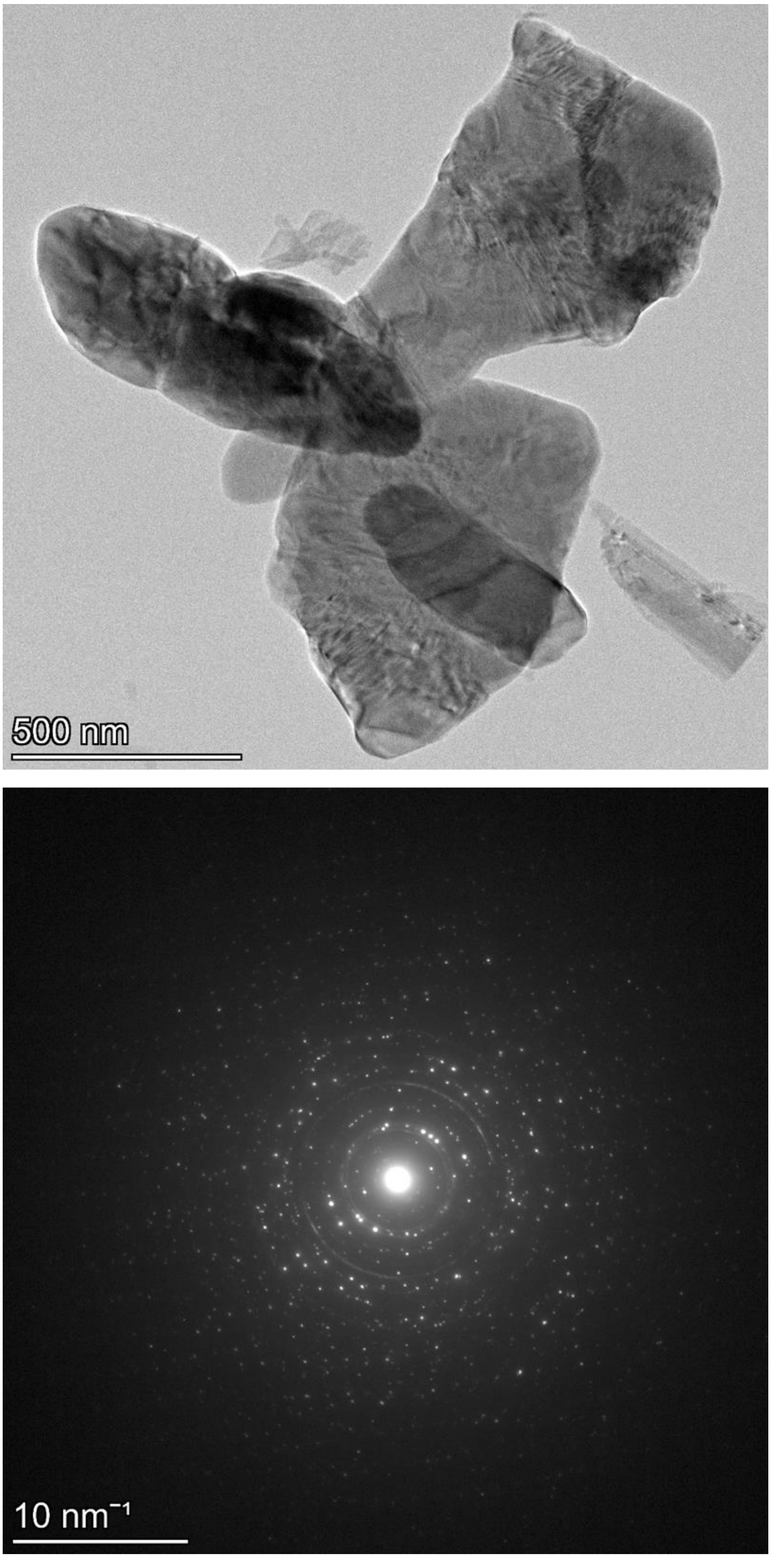

Figure S2. TEM images of the as-prepared KV_3O_8 powder and their diffraction pattern.

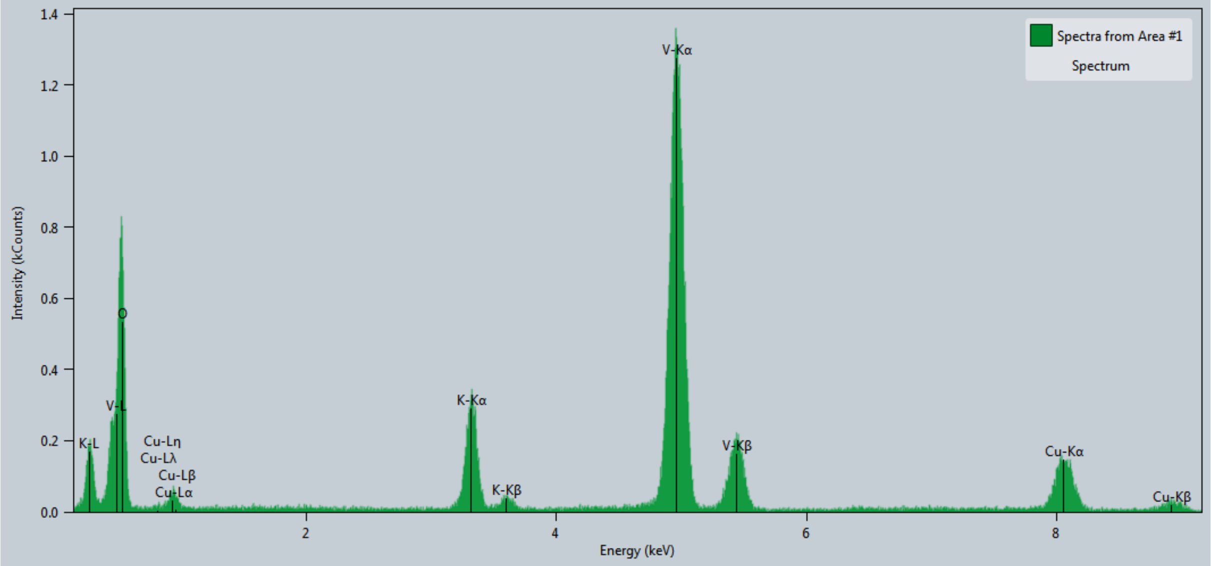

Figure S3. TEM–EDX spectra of pristine KV_3O_8 powder.

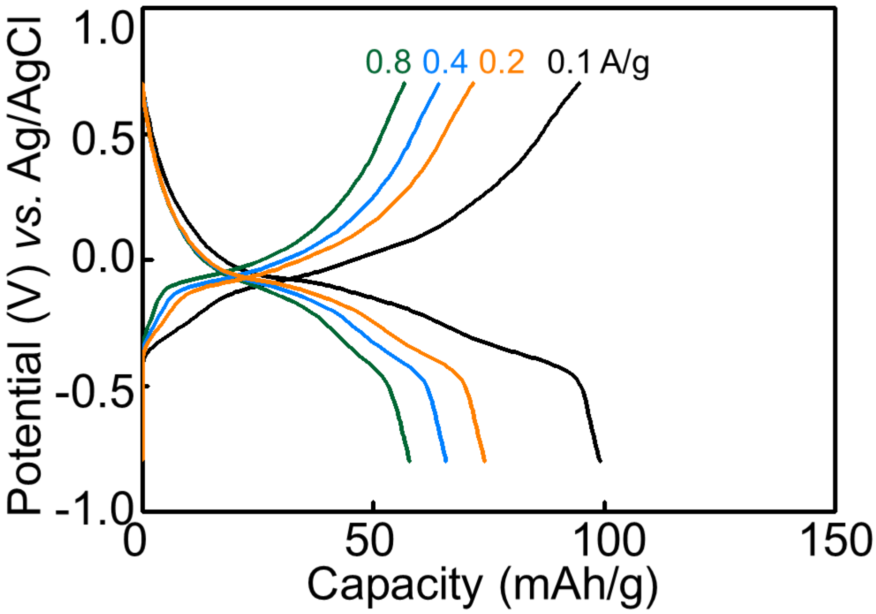

Figure S4. Galvanostatic charge/discharge curves of KV_3O_8 at various current densities.

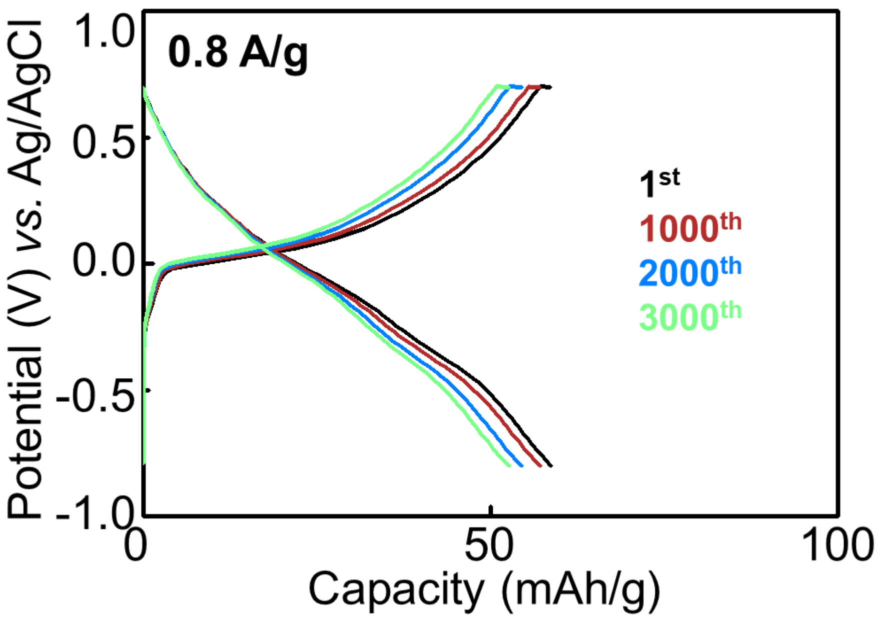

Figure S5. Galvanostatic charge/discharge curves of KV_3O_8 during the cycle performance at a constant current density of 0.8 A g^{-1}.

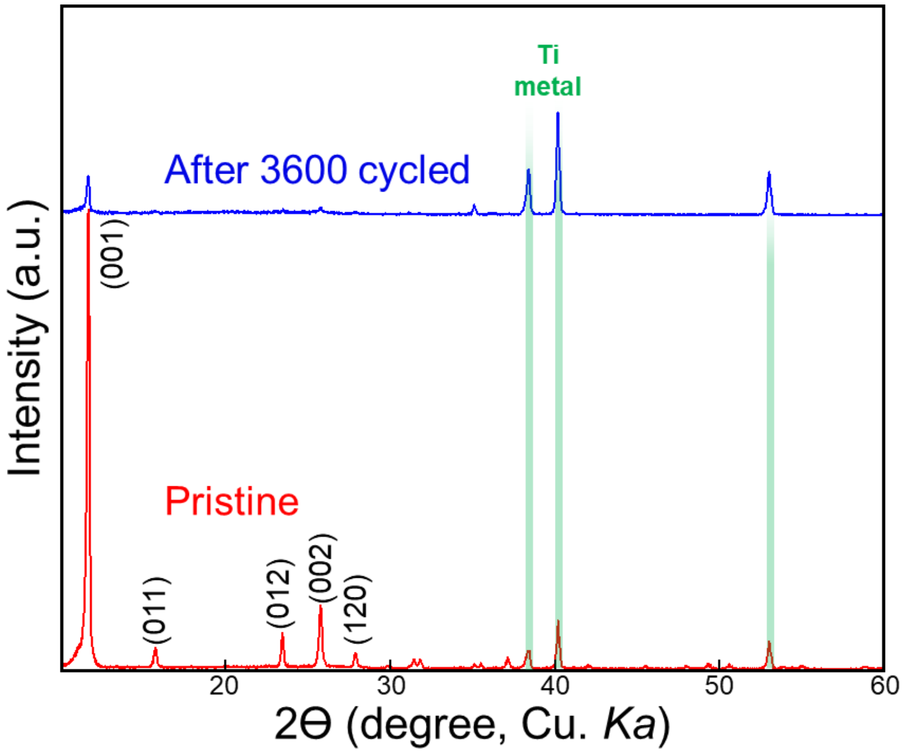

Figure S6. XRD patterns of pristine electrode and cycled KV_3O_8 electrodes after 3600 cycles.

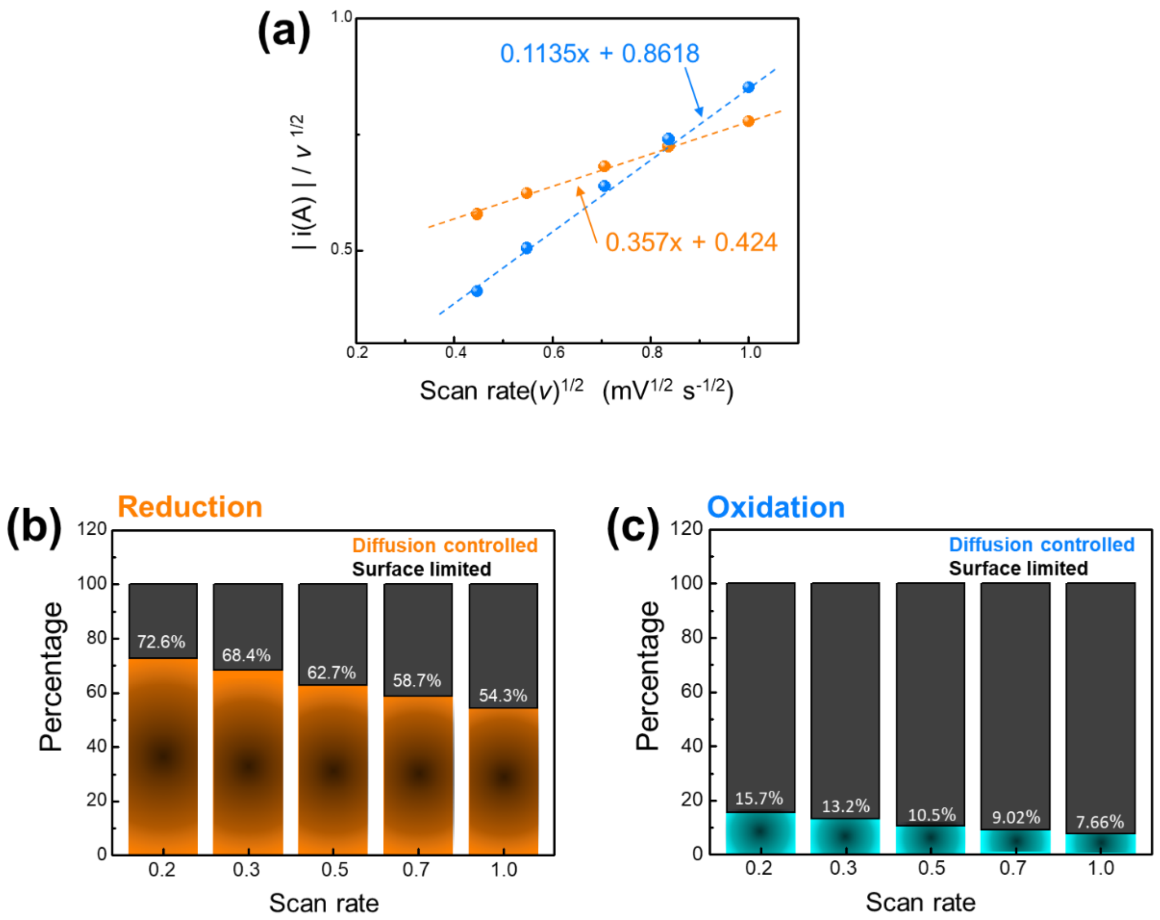

Figure S7. (a) Dependence of cathodic and anodic peak currents on the scan rate, which is used to evaluate the contributions of capacitive and intercalation mechanisms to energy storage. Calculated intercalation-to-adsorption ratios at various scan rates for (b) reduction (discharge) and (c) oxidation (charge) processes of the KV_3O_8 electrode.

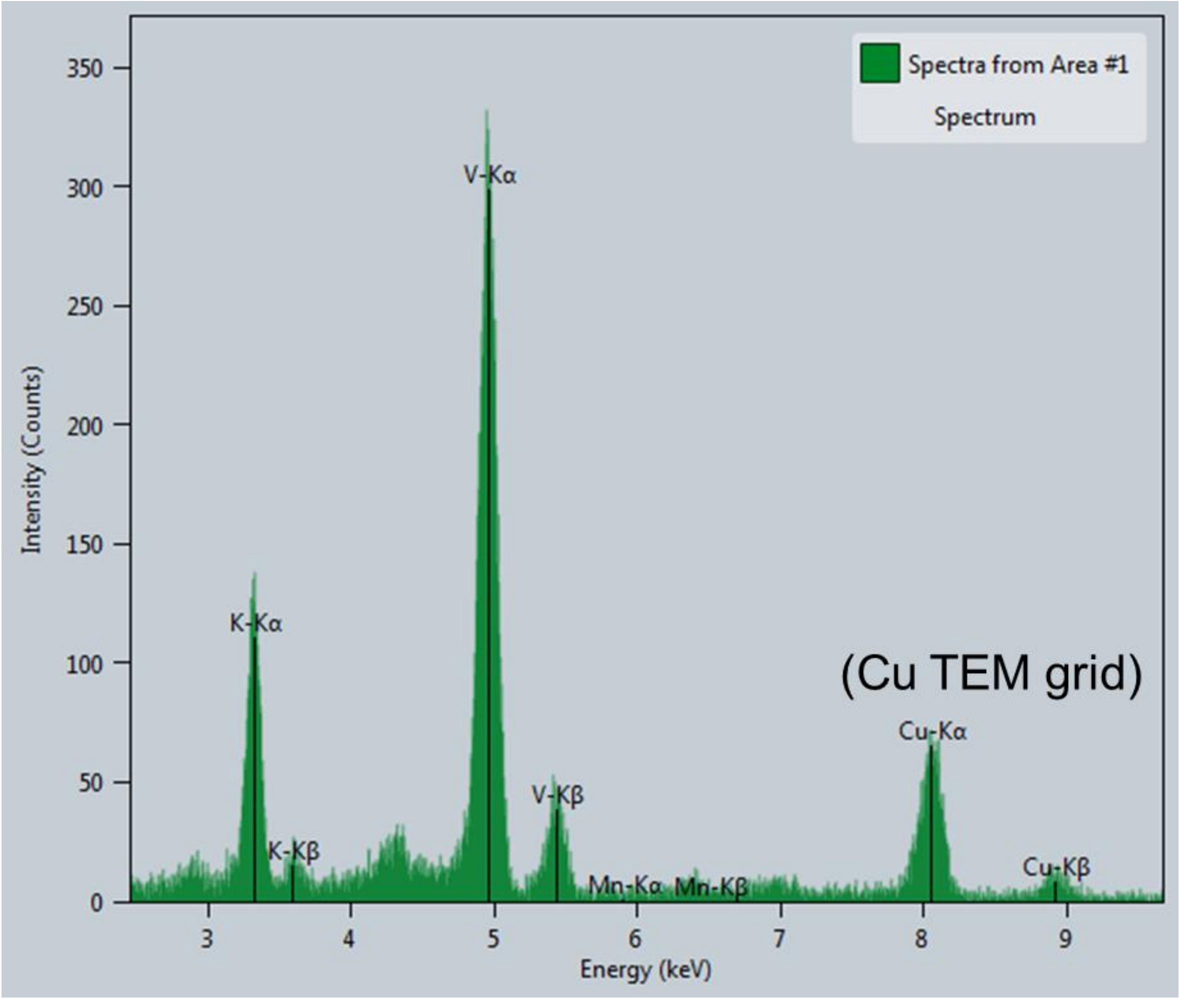

Figure S8. TEM–EDX spectra of the pristine KV_3O_8 electrode.

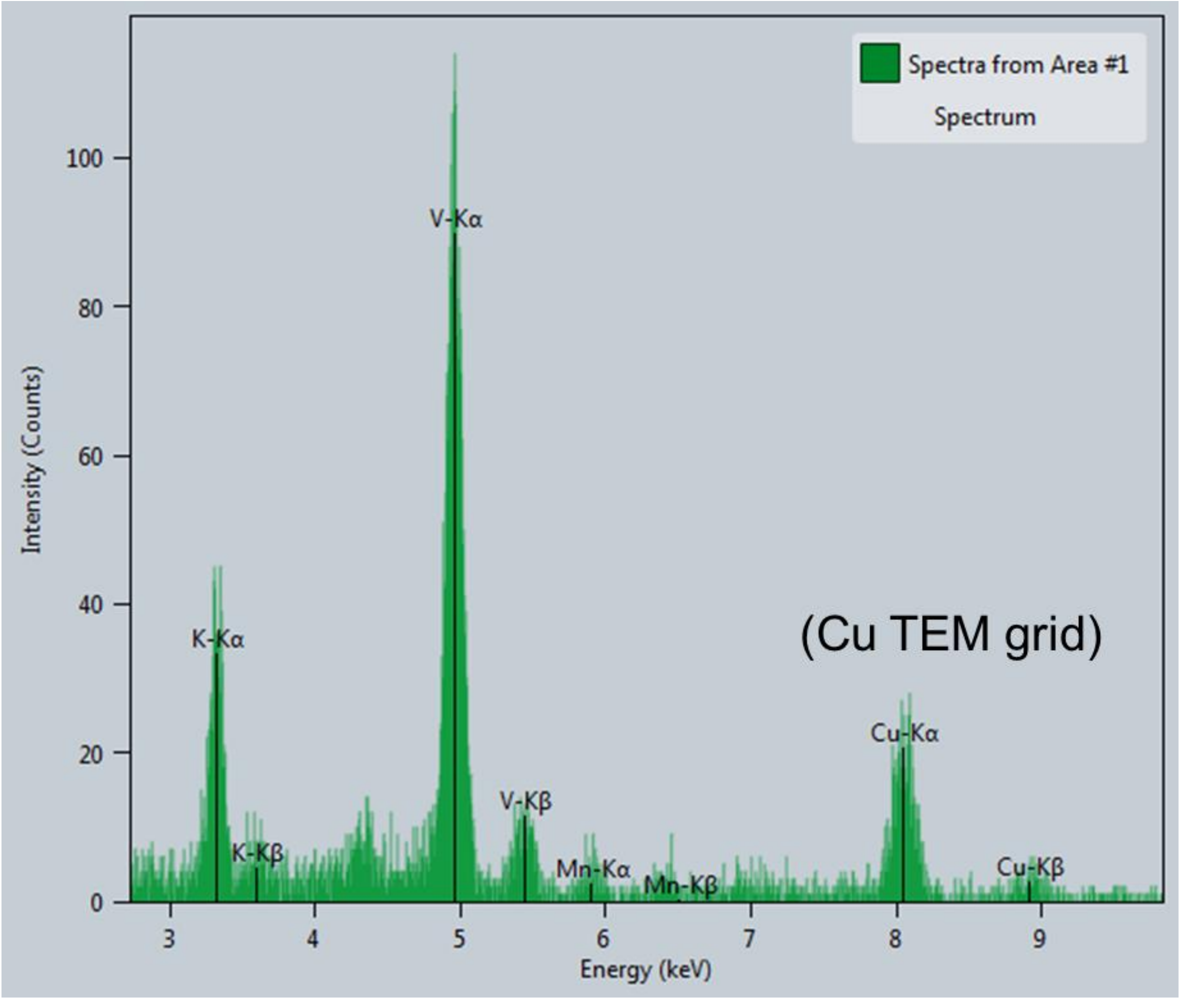

Figure S9. TEM–EDX spectra of the discharged KV_3O_8 electrode.

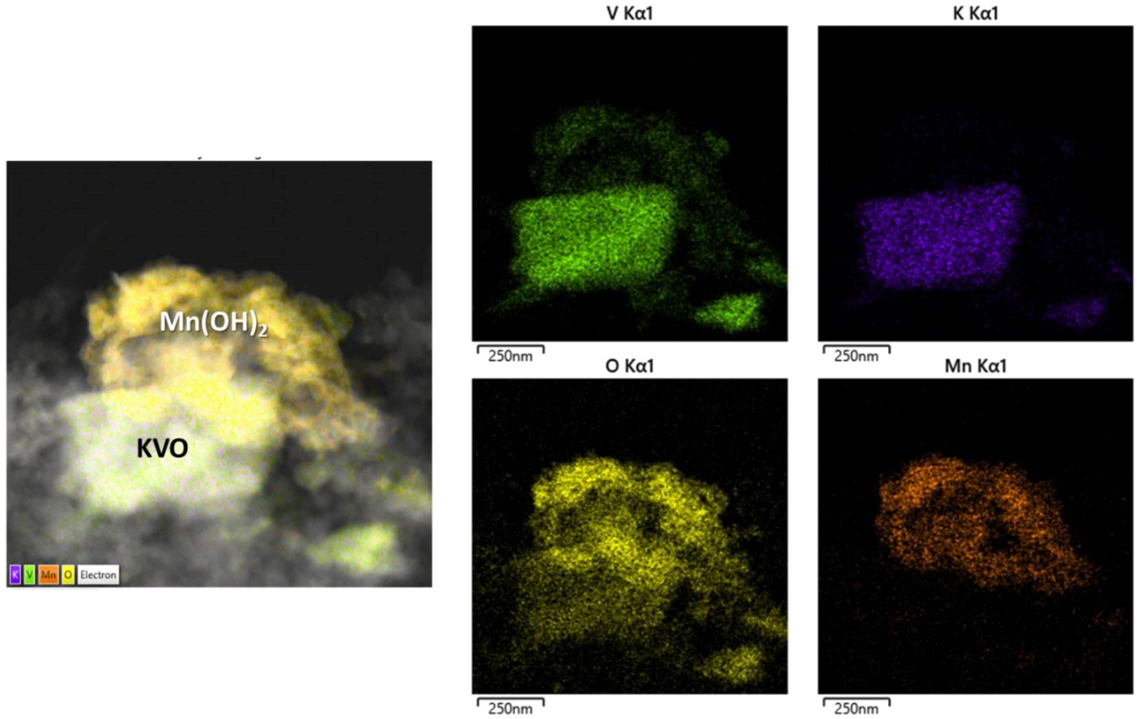

Figure S10. TEM–EDX elemental mapping of the re-charged KV_3O_8.

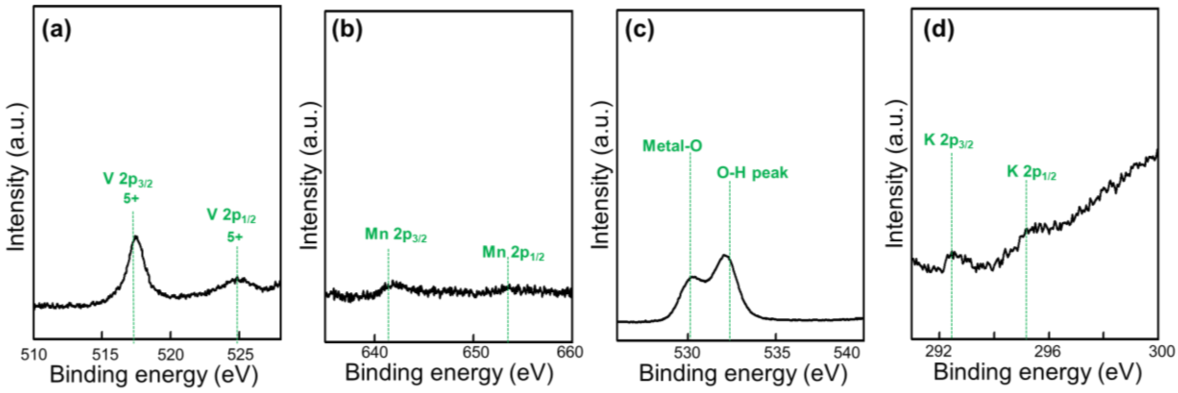

Figure S11. XPS spectra of KV_3O_8 at the re-charged states, showing the evolution of (a) V 2p, (b) Mn 2p, (c) O 1s, and (d) K 2p core levels.

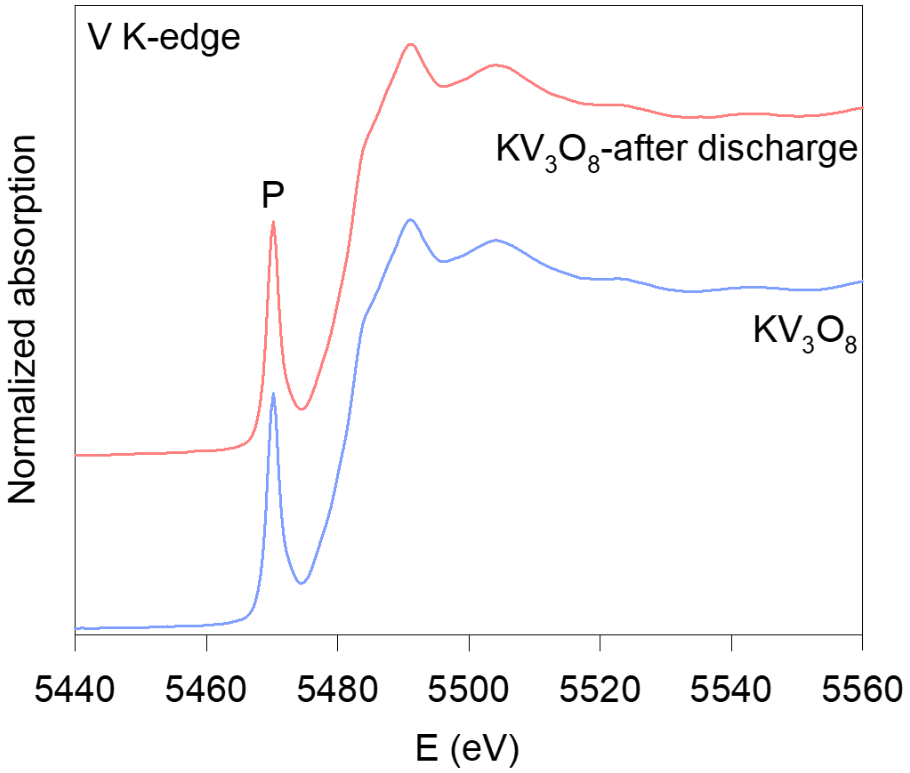

Figure S12. Ex situ V K-edge XANES spectra of KV_3O_8 before and after discharging process.

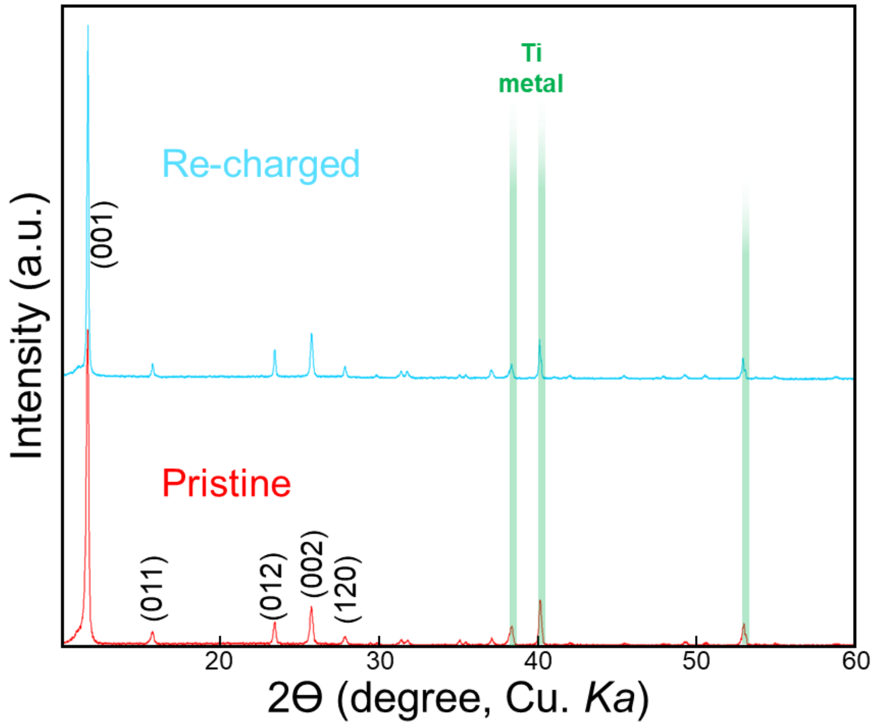

Figure S13. Ex situ XRD patterns of KV_3O_8 collected at the re-charged process.

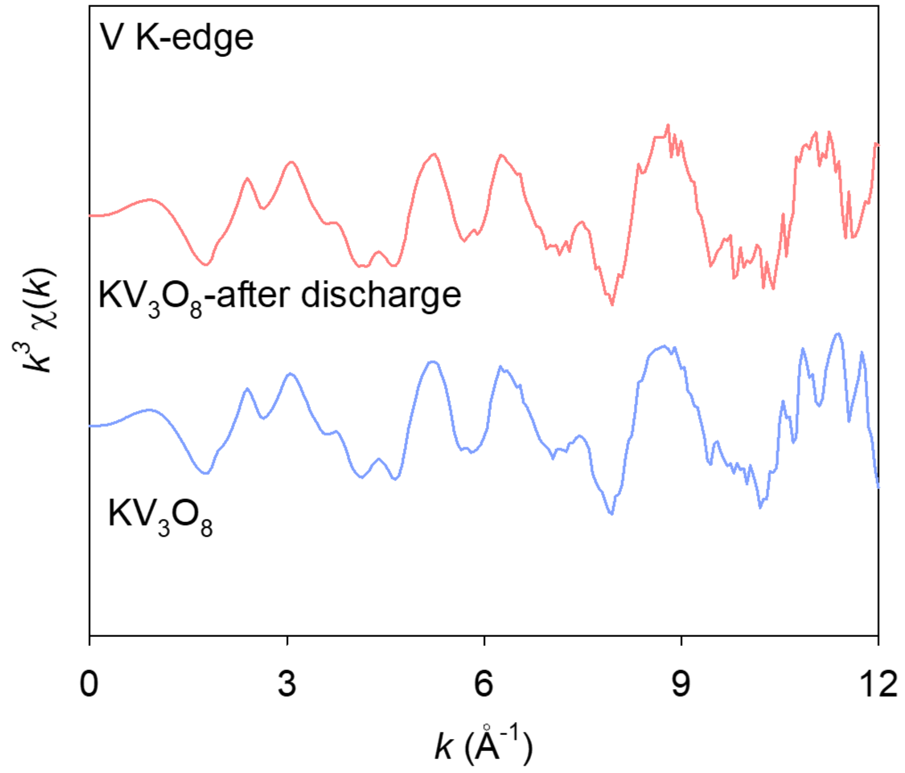

Figure S14. k^3-weighted V K-edge EXAFS spectra of KV_3O_8 before and after discharging process.

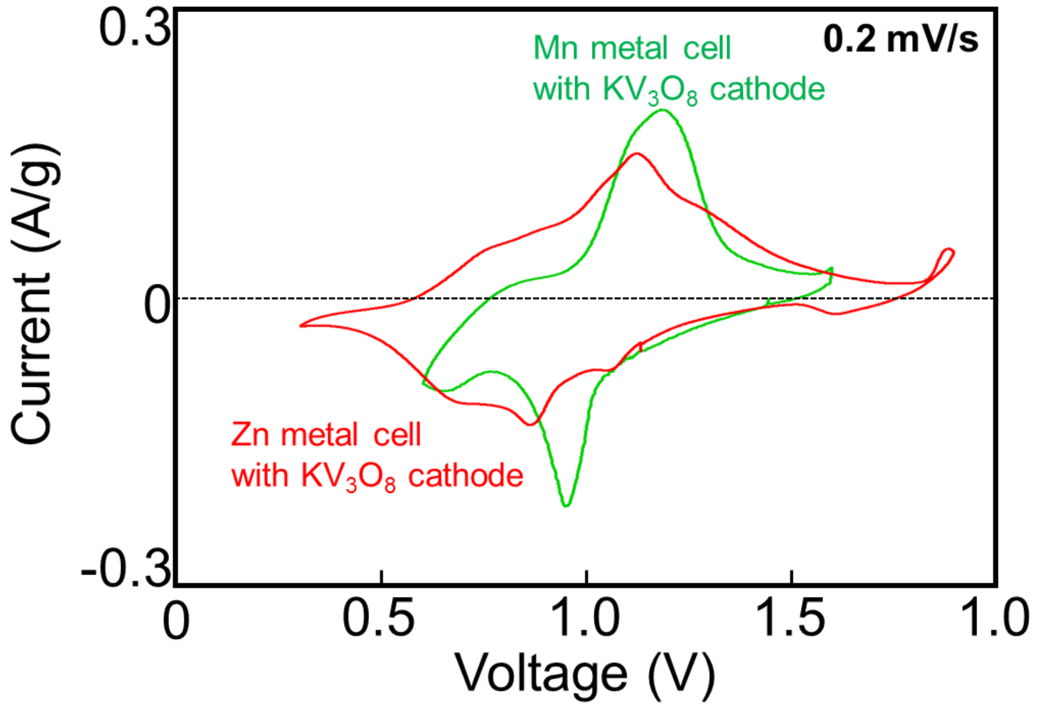

Figure S15. Cyclic voltammograms of KV_3O_8 with zinc (red) and manganese (green) metal anode.

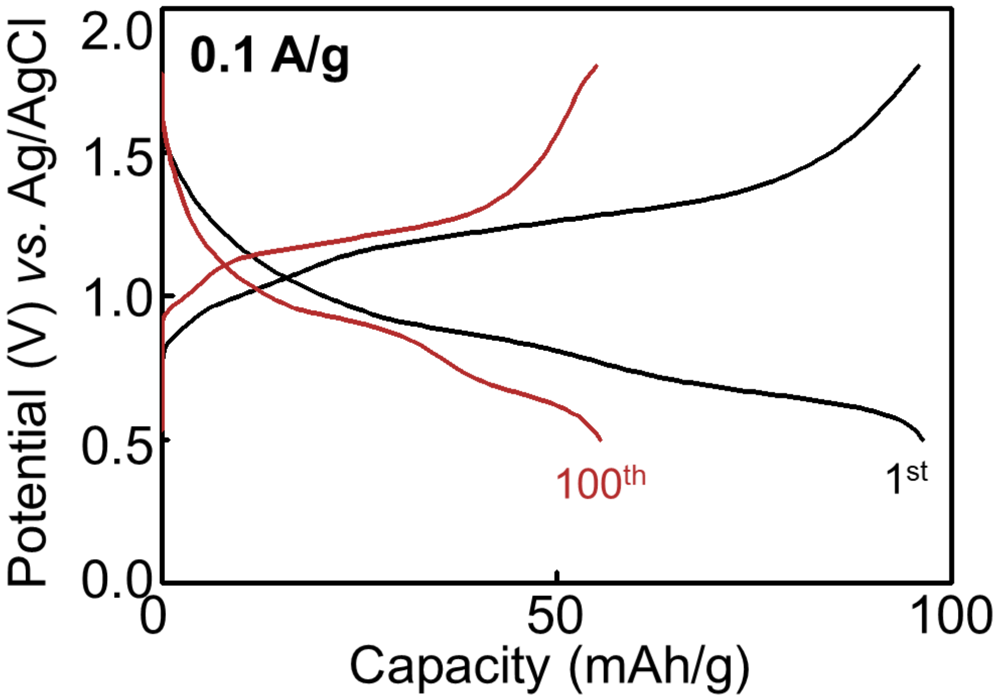

Figure S16. Galvanostatic charge/discharge curves of KV_3O_8 manganese metal battery at a constant current density of 0.1 A g^{-1}.
